# Depleting Tumor Cells Expressing Immune Checkpoint Ligands—A New Approach to Combat Cancer

**DOI:** 10.3390/cells10040872

**Published:** 2021-04-12

**Authors:** Fabrizio Marcucci, Cristiano Rumio

**Affiliations:** Department of Pharmacological and Biomolecular Sciences, University of Milan, Via Trentacoste 2, 20134 Milan, Italy; cristiano.rumio@unimi.it

**Keywords:** immune checkpoint, epithelial-mesenchymal transition, overexpression, ADC, bispecific, CAR T cells, effector functions, oncolytic virus, combination therapy

## Abstract

Antibodies against inhibitory immune checkpoint molecules (ICPMs), referred to as immune checkpoint inhibitors (ICIs), have gained a prominent place in cancer therapy. Several ICIs in clinical use have been engineered to be devoid of effector functions because of the fear that ICIs with preserved effector functions could deplete immune cells, thereby curtailing antitumor immune responses. ICPM ligands (ICPMLs), however, are often overexpressed on a sizeable fraction of tumor cells of many tumor types and these tumor cells display an aggressive phenotype with changes typical of tumor cells undergoing an epithelial-mesenchymal transition. Moreover, immune cells expressing ICPMLs are often endowed with immunosuppressive or immune-deviated functionalities. Taken together, these observations suggest that compounds with the potential of depleting cells expressing ICPMLs may become useful tools for tumor therapy. In this article, we summarize the current state of the art of these compounds, including avelumab, which is the only ICI targeting an ICPML with preserved effector functions that has gained approval so far. We also discuss approaches allowing to obtain compounds with enhanced tumor cell-depleting potential compared to native antibodies. Eventually, we propose treatment protocols that may be applied in order to optimize the therapeutic efficacy of compounds that deplete cells expressing ICPMLs.

## 1. Introduction

Immune checkpoint molecules (ICPMs) modulate innate or adaptive immune responses [1,2,3,4]. From a functional point of view, they can be divided into two broad classes: ICPMs that costimulate [4] and ICPMs that inhibit immune responses [1,2,3]. ICPMs form ligand-receptor pairs, with the receptors being predominantly expressed on immune cells and the ligands being predominantly expressed on antigen-presenting cells (APC), tumor cells or other cell types [5]. This distinction, however, is not absolute since ICPM receptors can be expressed also on tumor cells, while ICPM ligands (ICPMLs) can be expressed also on immune cells. For the purpose of this article and for the sake of clarity, we will refer to ICPMLs whenever these molecules are expressed on tumor cells and can serve as potential targets for cell-depleting compounds. Engagement of the receptor by the ligand gives rise to an inhibitory or stimulatory (costimulatory) signal to the immune cell. The number of ICPMs is constantly increasing as new molecules falling within one of the two functional classes are discovered.

From a molecular point of view, ICPMs belong to several families. Most ICPMs belong to the B7/CD28 or the tumor necrosis factor (TNF) superfamilies. The B7/CD28 family itself can be divided into three groups on the basis of phylogenetic analyses [3]. Group I includes B7-1 (CD80), B7-2 (CD86), CD28, cytotoxic T-lymphocyte antigen 4 (CTLA-4, CD152), inducible T-cell costimulatory (ICOS, CD278), and ICOS-ligand (ICOS-L, CD275). Group II includes programmed cell death protein 1 (PD-1, CD279), PD-ligand 1 (PD-L1, CD274), PD-L2 (CD273). Group III consists of B7-H3 (CD276), B7-H4, and human endogenous retrovirus-H long terminal repeat-associating protein 2 (HHLA2), transmembrane and immunoglobulin domain containing 2 (TMIGD2, CD28H). CD70 and CD137 ligand (CD137L) are members of the TNF superfamily (TNFSF7 and TNFSF9, respectively). CD40 is a member of the TNFR superfamily (TNFRSF5). CD47 and CD155 are members of the immunoglobulin (Ig) superfamily. Galectin-9 is an S-type lectin.

ICPMs play important roles in all types of immune responses, including those occurring during tumorigenesis. In fact, it is this role that has raised most interest from a therapeutic point of view because of the possibility to interfere with the activity of inhibitory checkpoints or exert agonistic activity on costimulatory immune checkpoints [5,6] and, by so doing, stimulating antitumor immune responses with the aim of delaying tumor progression or, optimally, leading to tumor eradication. As a result, several monoclonal antibodies (mAb) against inhibitory ICPMs, generally referred to as immune checkpoint inhibitors (ICIs), have received regulatory approval and have yielded favorable therapeutic effects in a significant fraction of patients affected by several tumor types [5,7,8]. Interestingly, tumor cells often express ICPMLs on a sizeable fraction of tumor cells and overexpress them compared to normal cells [9]. This behavior suggests the possibility of targeting these ICPMLs for therapeutic purposes.

In this article we summarize the role of tumor cell-associated ICPMLs in tumor biology as well as the approaches that are being pursued in order to obtain compounds that deplete tumor cells expressing ICPMLs. We will not address here neither the effects of ICPMs on antitumor immune responses nor the clinical results obtained so far with ICIs. There are excellent reviews that cover these aspects, several of which are cited throughout this article.

## 2. Mechanisms Underlying the Overexpression of ICPMLs on Tumor Cells

Overexpression of ICPMLs on tumor cells can be the result of different stimuli, either cell-autonomous stimuli or stimuli from the tumor microenvironment (TME). The mechanisms underlying the overexpression of ICPMLs on tumor cells have been most thoroughly investigated for PD-L1 and have been reviewed recently [9]. As regards tumor cell-autonomous stimuli, overexpression of PD-L1 can be the result of intrachromosomal or extrachromosomal events. Copy number alterations in chromosomal region 9p24.1 that encompasses the loci for PD-L1 and PD-L2, inversions, deletions, translocations, generation of chimeric fusion transcripts, and disruption or mutation of the 3′-untranslated region of the *PD-L1* gene are intrachromosomal events that can lead to PD-L1 overexpression [10,11,12]. Tumor cell-autonomous, extrachromosomal events are receptor-activating mutations or receptor overexpression [13], gain-of-function or loss-of-function mutations affecting intracellular signaling molecules [14,15], activation or overexpression of transcription factors (e.g., hypoxia-inducible factor-α, signal transducer and activator of transcription (STAT) 3, MYC) [16,17,18]. More recently, also epigenetic mechanisms have been reported to induce or contribute to the overexpression of tumor cell-associated PD-L1 [19,20]. Tumor cell-exogenous stimuli that can lead to the overexpression of PD-L1 are cytokines (e.g., interferon (IFN)-γ, tumor necrosis factor (TNF)-α) [21,22] and various other stimuli from the TME like hypoxia or pseudohypoxia [18,23], antitumor drugs (chemotherapeutics, targeted therapeutics) [24] or metabolites (e.g., lactate) [25]. While the mechanisms leading to the overexpression of other tumor cell-associated ICPMLs have been much less investigated, they appear to be similar to those for PD-L1. Thus, hypoxia or pseudohypoxia lead to the overexpression of B7-H4 [26], CD70 [27], CD47 [28]. Antitumor drugs lead to the overexpression of CD70 and B7-H3 [29,30]. Activation of the Ras-Raf-MEK-extracellular signal-regulated kinase pathway leads to overexpression of CD155 and CD137 [31,32], Hedgehog signaling to overexpression of CD155 [32], the Janus kinase 2-STAT3 pathway to overexpression of fibrinogen-like protein 1 (FGL1) [33]. While the stimuli that induce overexpression of ICPMLs on tumor cells appear to be similar, in some instances subtle differences in the intracellular signaling pathways regulating the expression of two different ICPMLs have been observed [34], suggesting that these differences may explain the different patterns of expression that have been observed between different tumor cell-associated ICPMLs (see Section 3).

## 3. The Consequences of the Expression of ICPMLs on the Biology of Tumor Cells

In addition to transmitting signals to other cells (mostly immune cells) upon engagement of their cognate receptors [3,8,35,36] tumor cell-associated ICPMLs also exert cell-autonomous functions. Thus, their expression is associated with changes whereby tumor cells acquire enhanced capabilities to migrate, invade and metastasize to distant organs, undergo faster growth and metabolic alterations [37,38], acquire tumor-initiating potential [28,29,39] as well as resistance to antitumor drugs and apoptosis [9]. Collectively, these changes, when they are accompanied by the expression of specific transcription factors and molecular modifications [40] are referred to as tumor cell epithelial-mesenchymal transition (EMT) [41,42]. Indeed, the causal relationship between ICPML expression on tumor cells and EMT has been shown in several instances with a variety of technical approaches (e.g., siRNA, CRISPR/Cas) [43,44,45,46,47]. Expression of ICPMLs on tumor cells can be both a consequence [48], as well as a cause of tumor cell EMT [43,44,49,50,51], suggesting the existence of a positive feedback loop between the expression of ICPMLs and EMT [9]. Interestingly, tumor cell EMT can also have immunosuppressive effects [52] and it has recently been shown that loss of the epithelial marker E-cadherin, a hallmark of EMT, reduces responsiveness to ICIs in a mouse melanoma model [53].

As regards individual ICPMLs, the following have been reported to be associated with tumor cell EMT: PD-1 [54,55] PD-L1 [54,55,56,57], PD-L2 [54,55], B7-H3 [55], B7-H4 [43,58], CTLA-4 [54,55], OX40 [54], CD47 [59,60,61], CD137 ligand [62], CD155 [44], FGL1 [46], T-cell immunoglobulin and mucin-domain containing-3 (TIM-3) [55,63] and B- and T-lymphocyte attenuator (BTLA, CD272) [55]. Other ICPMLs, while not having been formally associated with EMT (e.g., CD70, galectin-9), are expressed by tumor cells displaying EMT-related functionalities [64,65,66,67].

The data discussed so far suggest the existence of a close association between expression of ICPMLs on tumor cells and EMT and raise the question as to whether this association is absolute. In fact, data show that the association of an ICPML (PD-L1) and EMT on tumor cells is not coincident [39]. Moreover, as already mentioned, tumor cell-associated PD-L1 expression can be induced by intrachromosomal events. In these cases, PD-L1 overexpression is independent of tumor cell EMT [10,12,68], but it cannot be excluded that it may contribute to the induction of tumor cell EMT. The observation that genomic amplification targeting *PD-L1* and *PD-L2* is enriched in triple-negative breast cancer (TNBC), a cancer type with a predominantly mesenchymal phenotype suggests that this may, indeed, be the case [69].

Moreover, the lack of coincidence between ICPML expression and tumor cell EMT may also be the consequence of EMT plasticity, whereby tumor cells undergoing EMT cover a whole spectrum of phenotypes spanning from a fully epithelial to a fully mesenchymal one [70]. This suggests the possibility, for example, that an individual ICPML on tumor cells may be expressed at EMT initiation, when epithelial markers still predominate over mesenchymal markers. Moreover, heterogeneity of EMT marker expression is paralleled by the heterogeneity of ICPML expression [71]. Such heterogeneity applies both to individual ICPMLs, with ICPML-negative and -positive tumor cells coexisting within the same tumor [72], as well as to different ICPMLs showing non-overlapping or partially overlapping expression within the same tumor cell population. As regards the heterogeneous expression of different ICPMLs, it has been reported, for example, that a fraction of PD-L1-negative melanomas expressed high levels of CD155 and this was associated with a poor response to anti-PD-1/anti-CTLA4 therapy [73]. Moreover, expression of B7-H4 was prevalent among immune-cold TNBCs, and correlated inversely with that of PD-L1 [74,75]. In hepatocellular carcinoma tissues, FGL1 and PD-L1 had distinct distribution and relationships with each other [76]. Expression of Herpes virus entry mediator (HVEM) was found to be broader than that of PD-L1 on cells of melanoma metastases from 116 patients [77]. Moreover, in some situations, administration of an anti-ICPM antibody (anti-PD-1) has been shown to lead to the upregulation of an ICPML (TIM-3) [78].

## 4. Why Non-Depleting Antibodies Have Been Used against Inhibitory ICPMLs

Given the points discussed so far and, in particular, the close association between tumor cell expression of ICPML and an aggressive phenotype, it is somehow surprising to note that most of the ICIs against ICPMLs that are in current clinical use, have been selected so to be devoid of cell-depleting activity.

In fact, ICI antibodies of IgG1 isotype are able, in addition to inhibit the interaction with the cognate ICPM, to induce cytotoxic or phagocytic effects (antibody-dependent cellular cytotoxicity (ADCC), antibody-dependent cellular phagocytosis (ADCP), complement-dependent cytotoxicity (CDC)) on cells expressing the targeted antigen. As to currently used antibodies, the anti-PD-L1 mAb atezolizumab has an aglycosylated Fc region devoid of effector functions, and the anti-PD-L1 mAb durvalumab is of IgG1 isotype with three mutations in the Fc domain resulting in greatly reduced ADCC and CDC [79]. A notable exception to this picture is the anti-PD-L1 mAb avelumab, which will be discussed later. The reason as to why several clinically approved ICIs have been selected to be devoid of effector functions is due to the fact that ICPMLs can be expressed not only on tumor cells, but also on cells of the innate and adaptive immune system and that their depletion through ADCC, CDC or ADCP might lead to undesired immunosuppressive effects. In fact, taking a closer look to ICPML-expressing immune cells, one may reach the conclusion that their depletion may not be necessarily of harm, because in many instances such cells have immunosuppressive effects. In the following, we will briefly discuss this knowledge which has been obtained mainly for PD-L1.

Tumor-associated dendritic cells (DCs) upregulate PD-L1 in response to T-cell derived inflammatory cytokines like IFN-γ [80], while M1 macrophages do so in response to another inflammatory cytokine, interleukin (IL)-1β [81]. PD-L1^+^ DCs can lead to functional inactivation of T cells upon interaction with PD-1 [82]. Similarly, other PD-L1^+^ antigen-presenting cells like macrophages can induce anergy in T cells upon interaction with PD-1 [83], explaining why expression of PD-L1 on immune cells, rather than tumor cells, has been found in some studies to correlate with a favorable response to anti-PD-1 therapy [84]. Additionally, B7-H4 is expressed on immunosuppressive tumor-associated macrophages (TAM) [85]. Moreover, upon PD-1/PD-L1 interaction, macrophages can produce increased levels of immunosuppressive cytokines like IL-10, but reduced levels of inflammatory cytokines like IL-6 [83,86]. Additionally, tumor-infiltrating T cells can express PD-L1 upon activation and this PD-L1 is important for T-cell survival [87]. Ligation of T cell-associated PD-L1 can have immunosuppressive effects by promoting M2 polarization of macrophages, reducing the production of inflammatory cytokines and inducing an anergic phenotype or apoptosis in T-cells [88,89]. PD-L1 expression has also been documented on non-tumor cells of the TME that may play tumor-promoting and immunosuppressive roles like cancer-associated fibroblasts (CAF) [90]. Eventually, mice lacking CD155 on both tumor-infiltrating myeloid cells as well as tumor cells showed greater reduction of tumor growth and metastasis compared to mice lacking CD155 only on tumor cells [91]. Importantly, the immunosuppressive effects of ICPMLs may be context-dependent as has been shown for PD-L1, with tumor-associated PD-L1 playing a predominantly immunosuppressive role in some tumor types, and PD-L1 expressed on tumor-associated immune cells playing a predominantly immunosuppressive role in other tumor types [92,93]).

So far, we have listed several downsides related to ICPML expression on immune cells. There are, however, some observations suggesting that the expression of PD-L1 on immune cells may contribute to antitumor effects of the immune response. Thus, some tumors were shown to induce expression of PD-L1 on natural killer (NK) cells and this led to enhanced NK-cell function. These PD-L1-positive NK cells could be activated with an anti-PD-L1 antibody to perform increased antitumor activity [94]. Depletion of PD-L1-expressing NK cells led to the suppression of this antitumor mechanism. Still another possibility to be considered is that depletion of ICPML^+^ immunosuppressive cells triggers direct tumor-promoting effects of the immune system like those that may occur during hyperprogressive disease observed during ICI therapy [95,96,97].

## 5. Does Target Cell Depletion Contribute to the Therapeutic Activity of Some of the Approved ICIs?

There are several observations suggesting that currently used ICIs may exert their activity not only by inhibiting the transmission of inhibitory signals in immune cells. Thus, it has been shown that increasing tumoral PD-L1 expression may be predictive of a favorable response to PD-1 inhibition [98]. This observation is somehow at odds with the assumption that inhibition of the interaction between ICPML-ICPM receptor pairs interrupts only the transmission of a negative signal to immune cells. In fact, in this case the degree of inhibition should depend on the level of expression of the ICPM receptor on immune cells and its degree of occupancy by the anti-PD-1 ICI, while being relatively independent of the level of tumor cell-associated ICPML expression (in this case PD-L1). Rather, it suggests that inhibition of the interaction may also interrupt the transmission of a signal towards tumor cells, perhaps a signal that promotes EMT in tumor cells. However, if this would be the only mode of action of ICIs, then the association between tumoral PD-L1 expression and therapeutic efficacy should be absolute, and this is not the case. In fact, patients with low or (apparently) negative PD-L1 expression may respond to treatment [99] and, vice versa, not every patient with demonstrable tumoral PD-L1 expression responds to treatment [100]. Altogether, these results suggest that the therapeutic outcome following treatment with ICIs may be the result of two, possibly overlapping, activities: first, inhibition of the transmission of negative signals to immune cells; second, inhibition of the transmission of positive signals to tumor cells. At present, however, it is unclear to what extent the interruption of such positive signaling in tumor cells contributes to the therapeutic activity of ICIs currently used in the clinics.

These considerations, however, suggest that the use of a therapeutic compound that depletes ICPML^+^ tumor cells and ICPML^+^ immunosuppressive immune cells, might have some advantages compared to presently used ICIs. In fact, some preclinical studies in a mouse model have confirmed this assumption for anti-PD-L1 antibodies [101].

As briefly mentioned before, one ICI in current clinical use, the anti-PD-L1 avelumab, has preserved effector functions and, therefore, the potential of depleting PD-L1^+^ target cells. In fact, avelumab is an IgG1 mAb that promotes ADCC on PD-L1-positive cells of different tumor types both in vitro as well as in vivo [102,103,104]. In the clinics, avelumab has yielded durable responses in advanced Merkel cell carcinoma patients with an adverse event profile comparable to other ICIs [105]. On the basis of our current knowledge, however, it is fair to say that we do not know to what extent the preserved effector functions of avelumab play a role in the overall therapeutic efficacy of this ICI.

In addition to avelumab, other anti-ICPML mAbs with preserved effector functions are at various stages of preclinical or clinical development. These include antibodies against CD40 [106], CD47 [107,108], PD-L1 [109], CD70 [110] and B7-H4 [111]. Also in these cases, for all the more reason, it is presently impossible to estimate the contribution of the effector functions to the (potential) therapeutic efficacy of these antibodies.

## 6. Improving the Efficacy of Depleting Compounds by Enhancing the Expression of Tumor Cell-Associated ICPMLs

While data on the therapeutic efficacy of ICIs with preserved effector functions are still limited, available data on the anti-PD-L1 mAb avelumab suggest that their efficacy may not drastically increase compared to ICIs devoid of such functions. If this will be confirmed in future studies with the same or other antibodies, then one is led to ask if there are possibilities to improve the efficacy of mAbs endowed with cell-depleting potential. Available knowledge suggest that this goal may be achieved by means of two, non-mutually exclusive possibilities: first, enhancing the expression of tumor cell-associated ICPMLs; second, endowing antibodies with enhanced cell-depleting potential compared to native antibodies. In this section we address the first of these two possibilities. Given the non-coincident expression of different ICPMLs on tumor cells, the following discussion may not automatically apply to all tumor cell-associated ICPMLs. For this purpose, parallel studies, investigating several ICPMLs at the same time would be required. To our knowledge, these studies are not yet available.

The first and best-known class of molecules that upregulate the expression of tumor cell-associated ICPMLs are cytokines. IFN-γ stands out as the best known [81,102,112], but also other inflammatory cytokines like TNF-α, IL-6 and IL-1β have been shown to upregulate ICPMLs [113]. It is to note, however, that not all ICPMLs are upregulated by the same set of cytokines. B7-H4, for example, is upregulated in tumor cells by immunosuppressive cytokines like TGF-β1 or IL-10, but not by inflammatory cytokines like IFN-γ or TNF-α [114,115]. The systemic administration of cytokines, however, is precluded because of their toxicity and/or immunosuppressive effects, but approaches for the targeted delivery of these cytokines to the TME have been described [116,117] and may be investigated also for their potential to upregulate individual ICPMLs.

A second class of molecules to be considered for the upregulation of ICPMLs are, intriguingly, ICIs themselves. Thus, it has recently been reported that melanoma patients treated with anti-PD-1 antibodies greatly upregulate the expression of tumor- and macrophage-associated PD-L1 within days of commencing treatment [118]. Such upregulation may be, at least in part, the consequence of increased IFN-γ production in response to the anti-PD-1 antibody [119]. This suggests the possibility to pursue a two-step approach. First, treating patients with an ICI. Subsequently, when expression of the targeted tumor cell-associated ICPML has reached its zenith, using a second, depleting compound in order to target the largest possible number of tumor cells.

Not surprisingly, also chemotherapeutic drugs, a notorious stimulus for EMT induction [120], have been found to increase tumor cell-associated PD-L1 expression. This has been observed in preclinical models [121] and in metastatic CRC patients who received neoadjuvant chemotherapy [20]. CD155 is another ICPML that has been shown to increase in response to some, but not other chemotherapeutics [122,123]. It should be noted, however, that there are also reports claiming that tumor cell-associated PD-L1 expression decreases after (neoadjuvant) chemotherapy [124]. The reasons for these discrepancies are unclear.

Still another approach to enhance the expression of tumor cell-associated ICPMLs is to act on intracellular signaling pathways that are involved in their positive or negative regulation. Thus, tumor cell-associated PD-L1 expression was increased upon inhibition of cyclin-dependent kinases (CDK) 4/6 by blocking ubiquitination-mediated PD-L1 degradation [125]. Treatment of tumor-bearing mice with the CDK4/6 inhibitor palbociclib and an anti-PD-1 antibody resulted in improved survival compared with either treatment alone. These favorable effects, however, could be due, at least in part, also to other antitumor immunity-promoting effects that have been observed with CDK4/6 inhibitors such as increased production of type III interferons and enhanced tumor antigen presentation [126]. These results show that increasing tumor cell-associated PD-L1 expression improves therapy with an ICI, but suggest that such an approach may be useful also in view of depleting ICPML^+^ tumor cells. Additionally, nutlin-3, a small molecule *cis*-imidazoline analog that blocks the interaction between mouse double minute 2 homolog and p53, was found to increase tumor cell expression of the ICPMLs B7-H3 and PD-L1 [34]. Inhibition of the MEK pathway with trametinib enhanced the expression of B7-H3 in non-small cell lung cancer (NSCLC) and bladder cancer cells [30].

Targeting epigenetic mechanisms like DNA methylation, histone deacetylation or expression of the enhancer of zeste homolog 2 (EZH2) is another approach for upregulating ICPML expression. An article already referred to [20] has shown that decitabine, an inhibitor of DNA methyltransferase 1 (DNMT1) induced, as expected, DNA hypomethylation, which led to increased PD-L1 expression as well as increased expression of immune-related genes and tumor infiltration of T cells [20]. Increased expression of PD-L1 as a result of DNA hypomethylation has also been observed by others [127]. The clinically approved histone deacetylase inhibitor panobinostat increased IFN-γ-induced expression of PD-L1 in multiple myeloma cells [128]. Inhibition of the neddylation pathway with pevonedistat (MLN4924) increased the expression of tumor cell PD-L1 both by increasing PD-L1 mRNA levels as well as by blocking PD-L1 degradation [129]. While these observations refer only to PD-L1 it is reasonable to predict that similar results will be observed also for other ICPMLs.

## 7. Improving the Efficacy of Anti-ICPML Compounds by Increasing Their Cell-Depleting Potential

In this section we discuss the different approaches that are being pursued in order to obtain anti-ICPML compounds with increased cell-depleting potential compared to native antibodies. Table 1 gives a synthetic view of the expression of ICPMLs that will be discussed in this section on cells of different tumor types, immune cells and, if applicable, also on other cells of the TME. Table 2 gives a synoptic view of the different approaches as well as examples of corresponding anti-ICPML compounds that have been described in the literature and/or are in clinical development. Interestingly, not only antibodies against inhibitory ICPMLs are being used for this purpose, but also antibodies against costimulatory ICPMLs such as anti-CD40 antibodies. In fact, given that we are dealing with cell-depleting antibodies, their specificity for inhibitory or costimulatory ICPMs is likely of less relevance than for classical ICIs like the anti-PD-1 mAbs nivolumab or pembrolizumab.

### 7.1. Antibodies with Increased Effector Functions or Direct Apoptotic Effects against ICPML^+^ Tumor Cells

A first approach is to increase the constitutive effector functions of antibodies, in particular ADCC. This can be achieved by different means such as the generation of afucosylated or otherwise glycoengineered antibodies [179,180] or by introducing mutations in the Fc antibody domain [181]. Several afucosylated anti-ICPML antibodies or antibodies with mutated Fc regions (e.g., anti-B7-H3, anti-CD70) have been described and some are now in clinical studies [130,135,144].

Another class of antibodies induces apoptosis of tumor cells as a direct result of antigen engagement (in this case the ICPML), independently of their effector functions. So far, such antibodies have been described for ICPMLs like CD40, CD47 and galectin-9 [64,142,171,172,173,174]. At present, however, the potential efficacy of these antibodies in a clinical setting is unclear and they appear far from therapeutic use.

Antibody-drug conjugates (ADC) are composed of a mAb which is conjugated, through a cleavable or uncleavable linker, to a cytotoxic drug. Cleavable linkers have the potential advantage of releasing the drug after encounter of the antibody with the target antigen and, thereby, exerting a bystander effect on antigen-negative cells. This may represent an important advantage for ICPMLs which, in most cases, are expressed only on a variable fraction of the tumor cell population. Several ADCs have gained regulatory approval and many others are in clinical development [182,183]. Anti-ICPML ADCs against several ICPMs (e.g., PD-L1, CD70, B7-H3, B7-H4, TIM-1) have been reported and some are in active clinical development while the development of some others has been discontinued because of marginal single-agent activity or unacceptable toxicity [131,139,145,146,147,148,149,150,151,152,153,154,155,156].

### 7.2. Recruiting T-Cells for Depleting ICPML^+^ Tumor Cells

Bispecific antibodies or antibody fragments encompass two binding arms for two different antigens [184]. The bispecific antibodies used for the present purpose comprise one binding arm against an ICPML and a second arm that targets a molecule (e.g., CD3) expressed on T cells. Engagement of both arms brings the T cell in close proximity to the tumor cell in order to exert cytotoxic activity. T cell-engaging bispecific antibodies against several ICPMLs (CD47, CD155, B7-H3, B7-H4) have been described in the literature [30,157,158,159,160,161].

Chimeric antigen receptor (CAR) T cells are T cells engineered to express on the cell surface an antitumor antibody or antibody fragment, in this case an anti-ICPML antibody. These cells kill tumor cells upon antibody-driven recognition of the ICPML and subsequent activation of the cytotoxic mechanisms of the T cell. In some cases, other kinds of cytotoxic cells (e.g., NK cells) have been used for this purpose. As before, several CAR T cells have now been approved, while others are in clinical development [185]. As regards ICPMLs, CAR T cells against PD-L1 [162], B7-H3 [132,163,164,165], B7-H4 [166], CD47 [167], CD70 [168,169,170,186] have been described and some clinical studies with anti-ICPML CAR T cells have begun (Table 2).

### 7.3. Non-Antibody-Based Approaches for the Depletion of ICPML^+^ Tumor Cells

While antibodies have been, so far, the most popular recognition moiety for the targeting of ICPMLs, in principle they may be replaced with other specific recognition moieties which, however, are devoid of any constitutive effector function and rest solely on the therapeutic efficacy of the payload that may be linked to the ICPML binder. One of these recognition moieties are aptamers [187]. These are single-stranded oligonucleotides which can interact with desired targets with high affinity and specificity. One potential advantage of aptamers over antibodies is their small size and improved capacity in penetrating solid tumor tissues [187]. Enhanced tumor penetration leads both to increased therapeutic efficacy as well as reduced induction of drug resistance [188,189,190]. So far, an anti-PD-L1 aptamer conjugated to the cytotoxic drug paclitaxel has been described [175].

In one particular case, an ICPML has also been used as a target for an oncolytic virus. This is CD155, which functions also as poliovirus receptor. The neuroattenuated poliovirus strain PVSRIPO was engineered to replicate in and kill only tumor cells [176,177]. PVSRIP infection and tumor cell lysis induced also an inflammatory response that involved the recruitment of innate immune cells and, by so doing, promoted an adaptive antitumor immune response. PVSRIP has advanced into clinical investigations, and results from a phase I clinical trial [178] in patients with glioblastoma multiforme (GBM) have been published and appear promising.

## 8. Which Are the Most Promising ICPMLs as Targets for Cell-Depleting Compounds?

So far, we have discussed a large number of ICPMLs that can serve as targets for tumor cell-depleting compounds. At this point the question arises if there are some ICPMLs that appear more promising than others for this purpose. An optimal ICPML target should satisfy several criteria. First, it would be desirable to target an ICPML that is expressed on the largest possible number of tumor cells. Second, it would be even more advantageous if these molecules are expressed also on tumor accessory cells involved in supporting tumor growth. Third, while it is not realistic to identify a target that is completely absent on any type of normal cells, its expression on normal cells should be significantly lower than on tumor cells, so to allow an acceptable therapeutic index when targeting these molecules with cell-depleting compounds. Given these criteria, B7-H3 is certainly an interesting target. It is expressed not only on tumor cells but, in certain tumor types [130], also on tumor endothelial cells. In preclinical models, the use of an anti-B7-H3 ADC carrying a cytotoxic drug that caused depletion of both tumor cells and tumor endothelial cells, led to complete eradication of established tumors [131]. As regards its expression on tumor cells, it has been reported that in B7-H3^+^ pediatric solid tumors, almost all tumor cells were positive for this marker [164]. Eventually, moderate expression of B7-H3 on normal tissues did not seem to entail unacceptable toxicities suggesting that the therapeutic index was sufficiently broad [132]. Interestingly, B7-H3 has recently been shown to be expressed highly and homogeneously on cells of a lymphoma subtype and, for this reason, has been chosen as target for CAR T cells [158]. In fact, compared to solid tumors, hematologic malignancies offer advantages in terms of penetration of antitumor drugs, a problem that is particularly relevant for solid tumors and high molecular weight drugs or CAR T cells [188,189]. Given these observations, it is perhaps not surprising to note that B7-H3 is one of the most frequently chosen ICPML for the generation of tumor cell-depleting compounds (Table 2). CD70 is another popular ICPML target for several cell-depleting compounds, including CAR T cells (Table 2). Additionally, CD70 is expressed on a broad spectrum of solid tumors and hematologic malignancies [135]. While the expression of CD70 on normal cells has been reported to be modest, a note of caution, however, comes from results that have been reported with two anti-CD70 ADCs, which showed modest single-agent activity and, even more importantly, in one case were also accompanied by thrombocytopenia of high frequency and severity [146,147,148].

## 9. Conclusions and Perspectives

The therapeutic use of cell-depleting anti-ICPML compounds may have considerable advantages compared to other tumor cell-depleting approaches, but may also be burdened by limitations. In this final section we will discuss potential advantages and limitations as well as propose a possible treatment schedule that may maximize the advantages of this therapeutic approach.

A considerable advantage to be expected from using these compounds is that, as discussed in the previous sections, ICPML-expressing tumor cells are endowed with tumor-initiating and immunosuppressive potential, propensity to metastasize, and give rise to drug-resistant tumor cells. Moreover, their potential to deplete also immune cells expressing the same ICPML does not necessarily represent an inconvenience since most of these cells are mediators of immune deviation and/or suppression. Whether depletion of immunosuppressive cells may lead, in a way not dissimilar to what has been observed with ICIs, to autoimmune events, is a possibility that should be considered [191].

As of today, too little data obtained in clinical studies with compounds of this class are available to draw any conclusion. The observation that, in some circumstances, ICPML-expressing immune cells have antitumor properties needs to be considered. At present it appears difficult to predict when and how this could tip the balance towards immune evasion/suppression following the administration of compounds that deplete ICMPL^+^ cells. In fact, such a situation may not be dissimilar to what is observed with ICIs in some patients who respond to these therapies with accelerated tumor progression [95,96,97]. This, however, might be an acceptable price to pay if it occurs in a minority of patients while a broader audience of patients take advantage of the therapeutic efficacy of these compounds. Another possible limitation of compounds targeting tumor cell-associated ICPMLs is the fact that, in most cases, they target a subpopulation of tumor cells, even if these are the most aggressive ones. In fact, given the close association between ICPMLs and EMT, and given the plasticity of tumor cell EMT, it is reasonable to predict that, after depletion of ICPML^+^ tumor cells, ICPML^-^ tumor cells may give rise to new ICPML^+^ tumor cells if the same or similar cell-autonomous stimuli or stimuli from the TME persist [120]. In other words, approaches aimed at depleting ICPML^+^ tumor cells will unlikely be conclusive after a single treatment or a single cycle of treatments.

While the delivery of several cycles of therapy over prolonged periods of time is a common practice in tumor therapy it is, nevertheless, desirable, to limit the number of therapy cycles as much as possible in order to achieve clinical responses or, optimally, tumor eradication. For this purpose, one may combine a therapy aimed at depleting ICPML-expressing tumor cells with other therapies that target also ICPML^-^ tumor cell populations. The most obvious choices for such complementary therapies are cytotoxic drugs that act on drug-sensitive tumor cells which are expected to be of predominantly epithelial phenotype and ICPML^−^. Another possibility is to combine compounds targeting different ICPMLs. In a previous section, we have discussed that ICPMLs do not necessarily embrace the same tumor cell population(s) and the combination of compounds against different ICPMLs may yield a broader depletion than either compound alone. Such an approach may also be useful to contrast the compensatory upregulation of other ICPMLs that is observed in some instances following therapy with one ICI [78,192]. Still another possibility is to use compounds which enhance the expression of a given ICPML on tumor cells and to administer the anti-ICPML compound at the time of greatest expression of the ICPML. In Section 5 we have discussed several compounds having this potential.

Whether other combination therapies that are proving successful with ICIs, such as the combination of atezolizumab and the anti-vascular endothelial growth factor mAb bevacizumab [193,194] or the combined inhibition of PD-L1 and transforming growth factor-β [195,196], will prove successful also when using predominantly ICPML^+^ cell-depleting, rather than blocking compounds remains to be investigated.

**Figure 1:**
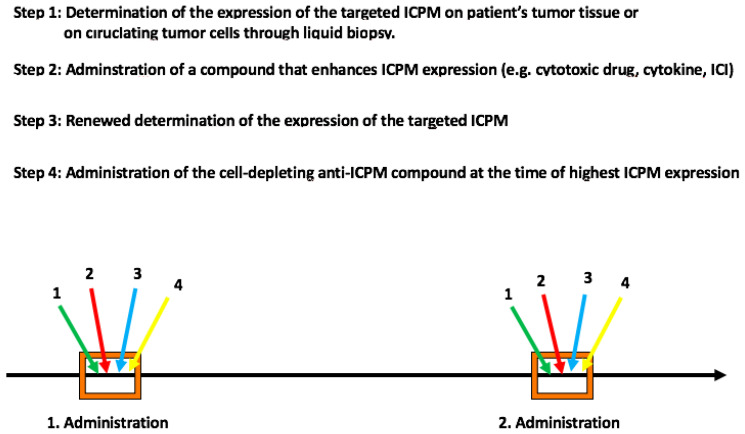
A four-step approach for optimal administration of cell-depleting anti-ICPM compounds. The figure depicts four steps that are proposed for the optimal administration of a cell-depleting anti-ICPM compound allowing to achieve the depletion of a maximum number of ICPM-expressing tumor cells. Abbreviations: ICI, immune checkpoint inhibitor; ICPM, immune checkpoint molecule.

Overall, we propose the following, four-step approach for the administration of a cell-depleting anti-ICPML compound (Figure 1). First, determination, before administration of the anti-IPCML compound, of the expression of the ICPML on tumor cells of the patients’ tumor tissue(s) or, even more desirably, on circulating tumor cells using a liquid biopsy approach. Second, administration of a drug known to enhance the expression of the targeted ICPML on tumor cells. Thereafter, renewed determination of ICPML expression on tumor cells, followed by administration of the ICPML-targeting compound at the time of greatest expression of the target. Administration will be repeated, according to a similar protocol, when tumor cells re-express the targeted ICPML on a sizeable fraction of tumor cells.

In conclusion, the possibility of using cell-depleting anti-ICPML compounds is certainly a promising avenue for obtaining new effective antitumor drugs. There is, however, still a significant road to go in order to gain a more complete picture of their therapeutic potential. Nevertheless, the location of these molecules at a crucial crossroad of tumor biology, involving tumor cells themselves, but also immune cells, suggest that it is worthwhile to explore the full potential of this approach.

## Figures and Tables

**Table 1:** Expression of ICPMLs on tumor cells, immune cells and other cells of the tumor microenvironment (TME).

ICPML	Tumor Type	Immune Cells	Other Cells of the TME	References
PD-L1	On cells of many hematologic and solid tumor types	Inducible expression on T cells, macrophages, DCs in response to inflammatory cytokines	On tumor endothelial cells, CAFs	[22,80,81,82,87,90]
B7-H3	On cells of many hematologic and solid tumor types	Inducible expression on T cells, NK cells, DCs and macrophages	On tumor endothelial cells, CAFs	[3,130,131,132,133]
B7-H4	On cells of many tumor types	Inducible expression on monocytes, macrophages, and myeloid DCs. Constitutive expression on TAMs, tumor T_regs_	On tumor endothelial cells, CAFs	[3,85,134]
CD70	On cells of many hematologic and solid tumor types, very frequent on RCC	On small subset of antigen-stimulated B and T cells, and mature DCs	On tumor endothelial cells, CAFs	[27,29,65,66,135,136,137]
CD155	On cells of many types of solid tumors	Low-level expression on immune cells, becomes up-regulated in response to inflammatory stimuli (LPS, cytokines). Expressed on tumor-infiltrating myeloid cells (macrophages, DCs).	On human vascular endothelial cells	[73,91,138]
TIM-1	O cells of many solid tumor types, most notably RCC and ovarian clear cell carcinoma	On T cells (in particular Th2), B_reg_ and DCs in mice.		[139]
CD47	On cells of many tumor types	Inducible (thrombospondin-1) expression on different types of immune cells (CD8^+^ T cells, macrophages, DCs, NK cells)	Ubiquitously expressed	[2,140]
Galectin-9	On cells of several human tumors including melanoma, multiple myeloma, mesothelioma	Constitutive expression on T_reg_, on Th cells upon activation, macrophages.		[64,141]
CD40	On cells of hematologic tumors	On APCs (DCs), B cells, monocytes		[142,143]

Abbreviations: APC, antigen-presenting cell; B_reg_, regulatory B cell; CAF, cancer-associated fibroblast; DC, dendritic cell; ICPML, immune checkpoint molecule ligand; LPS, lipopolysaccharide; NK, natural killer; PD-L1, programmed cell death protein-ligand 1; RCC, renal cell carcinoma; TAM, tumor-associated macrophage; Th, T helper; TIM-1, T-cell immunoglobulin and mucin domain 1; TME, tumor microenvironment; T_reg_, regulatory T cell.

**Table 2:** Anti-ICPML compounds with enhanced target cell-depleting potential reported in the literature and/or in clinical development.

Compound	Type of Construct	Preclinical or Clinical (ClinicalTrials.gov Identifier, Phase, Comments) Development	References
Antibodies with enhanced constitutive effector functions			
Anti-B7-H3 (eroblituzumab/MGA271)	mAb with mutated Fc domain	Clinical: NCT01391143, phase I; NCT02381314, phase I, plus ipilimumab; NCT02982941, phase I in children; NCT02475213, phase I, plus pembrolizumab; NCT02923180, phase II, neoadjuvant in prostate cancer; NCT04129320, NCT04634825, phase II/III, plus anti-PD-1 mAb or bispecific anti-PD-1xLAG-3 mAb.	[130]
Anti-CD70 (cusatuzumab/ARGX-110)	Afucosylated mAb	Clinical: NCT03030612, NCT04264806, NCT04241549, NCT04150887, NCT04023526, phase I/II, plus AZA or venetoclax in MDS, AML, CML; NCT02759250, phase I in NPC; NCT01813539, phase I/II neoplasms.	[135,144]
ADCs			
Anti-CD70 (SGN-75)	Humanized anti-CD70 mAb linked to tubulin inhibitor auristatin	Clinical: NCT01015911, phase I in NHL, RCC, modest single-agent activity; NCT01677390, phase Ib, plus everolimus in RCC.	[145,146]
Anti-CD70 (SGN-CD70A)	Anti-CD70 mAb linked to PBD dimer	Clinical: NCT02216890, phase I in NHL, RCC, showed modest single-agent activity and high frequency/severity of thrombocytopenia.	[147,148]
Anti-CD70 (BMS-936561/ MDX-1203)	Human anti-CD70 mAb linked to duocarmycin derivative	Clinical: NCT00944905, phase I in NHL, RCC.	[149,150]
Anti-B7-H3 (MGC018)	Humanized anti-B7-H3 mAb linked to duocarmycin	Clinical: NCT03729596, phase I/II, plus anti-PD-1 in several solid tumors.	[151]
Anti-B7-H3 (m276)	Human anti-B7-H3 mAb linked to PBD dimer	Preclinical: It depleted both B7-H3^+^ tumor cells as well as B7-H3^+^ tumor endothelial cells leading to the eradication of established tumors. Moderate expression of B7-H3 was detected also on normal tissues.	[131]
Anti-B7-H3	Anti-B7-H3 mAb linked to chlorin e6 for photodynamic therapy	Preclinical	[152]
Anti-B7-H4 (h1D11 TDC)	PBD linked to engineered cysteines of an anti-B7-H4 mAb via a protease-labile linker.	Preclinical: This ADC induced durable regression in different mouse models of TNBC.	[153]
anti-TIM-1 (CDX-014)	Human anti-TIM-1 linked to MMAE	Clinical: NCT02837991, phase I in RCC, development now discontinued.	[139]
Anti-PD-L1 (STM-108)	Mab anti-glycosylated PD-L1 linked to MMAE	Preclinical: Induced bystander killing on PD-L1^-^ tumor cells.	[154]
Anti-PD-L1	scFv-PD-L1 linked to the maytansinoid DM1	Preclinical: Specific binding to PD-L1^+^ tumor cells and antiproliferative activity in vitro.	[155]
Anti-PD-L1 (atezolizumab)	Atezolizumab linked to doxorubicin	Preclinical: Induced cell killing, disruption of tumor spheroids and induced apoptosis in a breast cancer cell line.	[156]
Bispecific Antibodies			
Anti-B7-H3xanti-CD3		Preclinical: MEK inhibitor trametinib augmented expression of B7-H3. Combined therapy (trametinib + bispecific mAb) increased T cell infiltration and significantly suppressed tumor growth.	[30]
Anti-B7-H3xanti-CD3		Preclinical: On hematological tumor cells, redirected T cells exhibited significant cytotoxicity, secreted more cytokines and granzyme B and expressed higher levels of activating marker CD69 compared to non-redirected T cells.	[157]
Anti-B7-H3xanti-CD3		Preclinical: On cells of ENKTCL redirected T cells killed tumor cells in vitro and suppressed the growth of NKTCL tumors in mouse models.	[158]
Anti-B7-H4xanti-CD3 (mAb clone #25xOKT3)	Two constructs: one Fab (anti-B7-H4) xscFv (anti-CD3),one scFvxscRv	Preclinical: In a humanized mouse model of breast cancer the bispecific Ab had strong antitumor activity and promoted the infiltration of CD8^+^ CTLs into the tumor without any adverse effects over the long term.	[159]
Anti-CD155xanti-CD3		Preclinical	[160,161]
CAR T or NK Cells			
Anti-PD-L1 CAR T cells	T cells expressing theextracellular domain of human PD-1 or the scFv of an anti-PD-L1	Preclinical: Induced regression of established PDAC cancer by >80% in both xenograft and orthotopic models.	[162]
Anti-B7-H3 CAR T cells (376.96 mAb)		Preclinical: Control of the growth of PDAC, ovarian cancer and neuroblastoma in xenograft mouse models and in a syngeneic tumor model without toxicity.	[132]
Anti-B7-H3 CAR T cells	scFv from an anti-B7-H3 mAb + PD-1 decoy receptor.	Preclinical: Potent antitumor activity in B7-H3^+^/B7-H1^+^ tumors in vivo.	[163]
Anti-B7-H3 CAR T cells	scFv derived from the anti-B7-H3 mAb enoblituzumab	Preclinical: Regression of established solid tumors in xenograft models. Efficacy dependent upon high surface antigen density on tumor tissues.	[164]
Anti-B7-H3 CAR NK cells (CAR-NK-92MI)		Preclinical: Inhibition of tumor growth in mouse xenografts of NSCLC and prolonged survival of mice.	[165]
Anti-B7-H4 CAR T cells		Preclinical: Inhibition of growth of B7-H4^+^ human ovarian tumor xenografts, but lethal toxicity was observed 6-8 weeks after therapy due to expression of B7-H4 in ductal and mucosal epithelial cells in normal tissues.	[166]
Anti-CD47 CAR NK cells	scFv from an anti-CD47 mAb	Preclinical: Inhibition of pancreatic xenograft tumor growth after intratumoral injection in mice.	[167]
Anti-CD70 CAR T cells	Anti-human and -mouse CD70 CAR T cells	Preclinical: Both human and mouse anti-CAR T cells induced regression of established GBM in xenograft and syngeneic models.	[168]
Anti-CD70 CAR T cells	Truncated CD27, the CD70 receptor, is the CD70 binder	Preclinical: Elimination of CD70-positive HNSCC cells.	[169]
Anti-CD70 CAR T cells	Truncated CD27, the CD70 receptor, was used as CD70 binder	Preclinical: Adoptively transferred anti-CD70 CAR T cells induced regression of established murine xenografts.	[170]
Anti-CD70 CAR T cells		Clinical: NCT04662294, phase I in AML, MM, NHL	N.A.
Anti-CD70 CAR T cells		Clinical: NCT03125577, NCT04429438, phase I/II in hematological B-cell malignancies.	N.A.
Anti-CD70 CAR T cells (CTX130)	Anti-CD70 allogeneic T cells.	Clinical: NCT04502446, phase I in relapsed or refractory T or B cell malignancies.	N.A.
Antibodies Inducing Cell Death Independently of Effector Functions			
Anti-CD40 mAb (dacetuzumab)		Preclinical: Dacetuzumab + anti-CD20 mAb rituximab gave synergistic apoptotic effects on NHL cells through distinct, but complementary apoptotic signal transduction pathways.	[142]
Anti-CD47 (mAb AO-176)		Clinical: NCT03834948, phase I/II, alone or with paclitaxel in solid tumors; NCT04445701, phase I/II alone or with bortezomib in MM.Preclinical: Induced tumor cell phagocytosis and cytotoxicity on human tumor cells but not normal cells.	[171]
Anti-CD47 mAb Ad22		Preclinical: Ad22 induced apoptosis of Jurkat cells and preactivated PBMC	[172]
Anti-CD47 (mAb CC2C6)		Preclinical: Soluble CC2C6 induced apoptosis of T-ALL cells, restored phagocytosis, and synergized with low-dose chemotherapeutics to induce apoptosis.	[173]
Anti-CD47 (mAb B6H12.2)		Preclinical: Enhanced phagocytosis of a set of human pancreatic CSCs and directly induced apoptosis in the absence of macrophages.	[174]
Anti-galectin 9 (mAb P4D2)		Preclinical: Induced MM cell apoptosis, inhibited tumor growth and reduced tumor infiltration of M2 macrophages.	[64]
Aptamers			
Anti-PD-L1 aptamer-drug conjugate	Aptamer-paclitaxel conjugate	Preclinical: The anti-PD-L1 aptamer inhibited PD-1/PD-L1 interaction and restored T-cell function. The conjugate inhibited proliferation of PD-L1-overexpressing TNBC cells.	[175]
Oncolytic Virus			
Oncolytic virus binding to CD155	Neuroattenuated poliovirus strain PVSRIPO that replicates in and kills only tumor cells	Clinical: NCT03564782, NCT03712358, NCT02986178, NCT03043391, NCT01491893, phase I/II in invasive breast cancer, melanoma, GBM; NCT04479241, NCT04577807, NCT04690699, phase II plus anti-PD-1or -PD-L1 in GBM, melanoma or other solid tumors.	[176,177,178]

Abbreviations: Ab, antibody; AML, acute myeloid leukemia; AZA, azacytidine; CAR, chimeric antigen receptor; CML, chronic myeloid leukemia; CSC, cancer stem-like cell; CTL, cytotoxic T-lymphocyte; ENKTCL, Extranodal nasal natural killer (NK)/T cell lymphoma; Fc, fraction crystallizable, GBM, glioblastoma multiforme; HNSCC, head and neck squamous cell carcinoma; LAG-3, lymphocyte-activation gene 3; mAb, monoclonal antibody; MDS, myelodysplastic syndrome; MM, multiple myeloma; MMAE, monomethyl auristatin E; NHL, non-Hodgkin lymphoma; NPC, nasopharyngeal carcinoma; NSCLC, non-small cell lung cancer; PBD, pyrrolobenzodiazepine; PBMC, peripheral blood mononuclear cells; PD-1, programmed cell death protein 1; PD-L1, PD ligand 1; RCC, renal cell carcinoma; scFv, single-chain fragment variable; T-ALL, T-cell acute lymphoblastic leukemia; TIM-1, TIM-1, T-cell immunoglobulin and mucin domain 1; TNBC, triple-negative breast cancer.

## Data Availability

No new data were created or analyzed in this study. Data sharing is not applicable to this article.

## References

[1] Sharpe A.H., Pauken K.E. (2018). The diverse functions of the PD1 inhibitory pathway. Nat. Rev. Immunol..

[2] Feng M., Jiang W., Kim B.Y.S., Zhang C.C., Fu Y.X., Weissman I.L. (2019). Phagocytosis checkpoints as new targets for cancer immunotherapy. Nat. Rev. Cancer.

[3] Janakiram M., Shah U.A., Liu W., Zhao A., Schoenberg M.P., Zang X. (2017). The third group of the B7-CD28 immune checkpoint family: HHLA2, TMIGD2, B7x, and B7-H3. Immunol. Rev..

[4] Watts T.H. (2005). TNF/TNFR family members in costimulation of T cell responses. Annu. Rev. Immunol..

[5] Pardoll D. (2012). The blockade of immune checkpoints in cancer immunotherapy. Nat. Rev. Cancer.

[6] Mayes P.A., Hance K.W., Hoos A. (2018). The promise and challenges of immune agonist antibody development in cancer. Nat. Rev. Drug Discov..

[7] Ribas A., Wolchok J.D. (2018). Cancer immunotherapy using checkpoint blockade. Science.

[8] Andrews L.P., Yano H., Vignali D.A.A. (2019). Inhibitory receptors and ligands beyond PD-1, PD-L1 and CTLA-4: Breakthroughs or backups. Nat. Immunol..

[9] Marcucci F., Rumio C., Corti A. (2017). Tumor cell-associated immune checkpoint molecules—Drivers of malignancy and stemness. Biochim. Biophys. Acta.

[10] George J., Saito M., Tsuta K., Iwakawa R., Shiraishi K., Scheel A.H., Uchida S., Watanabe S.I., Nishikawa R., Noguchi M. (2017). Genomic amplification of CD274 (PD-L1) in small-cell lung cancer. Clin. Cancer Res..

[11] Ota K., Azuma K., Kawahara A., Hattori S., Iwama E., Tanizaki J., Harada T., Matsumoto K., Takayama K., Takamori S. (2015). Induction of PD-L1 expression by the EML4-ALK oncoprotein and downstream signaling pathways in non-small cell lung cancer. Clin. Cancer Res..

[12] Kataoka K., Shiraishi Y., Takeda Y., Sakata S., Matsumoto M., Nagano S., Maeda T., Nagata Y., Kitanaka A., Mizuno S. (2016). Aberrant PD-L1 expression through 3’-UTR disruption in multiple cancers. Nature.

[13] Balan M., Mier y Teran E., Waaga-Gasser A.M., Gasser M., Choueiri T.K., Freeman G., Pal S. (2015). Novel roles of c-Met in the survival of renal cancer cells through the regulation of HO-1 and PD-L1 expression. J. Biol. Chem..

[14] Parsa A.T., Waldron J.S., Panner A., Crane C.A., Parney I.F., Barry J.J., Cachola K.E., Murray J.C., Tihan T., Jensen M.C. (2007). Loss of tumor suppressor PTEN function increases B7-H1 expression and immunoresistance in glioma. Nat. Med..

[15] Lastwika K.J., Wilson W., Li Q.K., Norris J., Xu H., Ghazarian S.R., Kitagawa H., Kawabata S., Taube J.M., Yao S. (2016). Control of PD-L1 expression by oncogenic activation of the AKT–mTOR pathway in non–small cell lung cancer. Cancer Res..

[16] Marzec M., Zhang Q., Goradia A., Raghunath P.N., Liu X., Paessler M., Wang H.Y., Wysocka M., Cheng M., Ruggeri B.A. (2008). Oncogenic kinase NPM/ALK induces through STAT3 expression of immunosuppressive protein CD274 (PD-L1, B7-H1). Proc. Natl. Acad. Sci. USA.

[17] Sun Y., Yu M., Qu M., Ma Y., Zheng D., Yue Y., Guo S., Tang L., Li G., Zheng W. (2020). Hepatitis B virus-triggered PTEN/β-catenin/c-Myc signaling enhances PD-L1 expression to promote immune evasion. Am. J. Physiol. Gastrointest. Liver Physiol..

[18] Noman M.Z., Desantis G., Janji B., Hasmim M., Karray S., Dessen P., Bronte V., Chouaib S. (2014). PD-L1 is a novel direct target of HIF-1α, and its blockade under hypoxia enhanced MDSC-mediated T cell activation. J. Exp. Med..

[19] Xiong W., Deng H., Huang C., Zen C., Jian C., Ye K., Zhong Z., Zhao X., Zhu L. (2019). MLL3 enhances the transcription of PD-L1 and regulates anti-tumor immunity. Biochim. Biophys. Acta Mol. Basis Dis..

[20] Huang K.C., Chiang S.F., Chen W.T., Chen T.W., Hu C.H., Yang P.C., Ke T.W., Chao K.S.C. (2020). Decitabine augments chemotherapy-induced PD-L1 upregulation for PD-L1 blockade in colorectal cancer. Cancers.

[21] Kondo A., Yamashita T., Tamura H., Zhao W., Tsuji T., Shimizu M., Shinya E., Takahashi H., Tamada K., Chen L. (2010). Interferon-gamma and tumor necrosis factor-alpha induce an immunoinhibitory molecule, B7-H1, via nuclear factor-kappaB activation in blasts in myelodysplastic syndromes. Blood.

[22] Lee Y., Shin J.H., Longmire M., Wang H., Kohrt H.E., Chang H.Y., Sunwoo J.B. (2016). CD44+ cells in head and neck squamous cell carcinoma suppress T-cell-mediated immunity by selective constitutive and inducible expression of PD-L1. Clin. Cancer Res..

[23] Messai Y., Gad S., Noman M.Z., Le Teuff G., Couve S., Janji B., Kammerer S.F., Rioux-Leclerc N., Hasmim M., Ferlicot S. (2016). Renal cell carcinoma programmed death-ligand 1, a new direct target of hypoxia-inducible factor-2α, is regulated by von Hippel-Lindau gene mutation status. Eur. Urol..

[24] Peng J., Hamanishi J., Matsumura N., Abiko K., Murat K., Baba T., Yamaguchi K., Horikawa N., Hosoe Y., Murphy S.K. (2015). Chemotherapy induces programmed cell death-ligand 1 overexpression via the nuclear factor-κB to foster an immunosuppressive tumor microenvironment in ovarian cancer. Cancer Res..

[25] Feng J., Yang H., Zhang Y., Wei H., Zhu Z., Zhu B., Yang M., Cao W., Wang L., Wu Z. (2017). Tumor cell-derived lactate induces TAZ-dependent upregulation of PD-L1 through GPR81 in human lung cancer cells. Oncogene.

[26] Jeon Y.K., Park S.G., Choi I.W., Lee S.W., Lee S.M., Choi I. (2015). Cancer cell-associated cytoplasmic B7-H4 is induced by hypoxia through hypoxia-inducible factor-1α and promotes cancer cell proliferation. Biochem. Biophys. Res. Commun..

[27] Ruf M., Mittmann C., Nowicka A.M., Hartmann A., Hermanns T., Poyet C., van den Broek M., Sulser T., Moch H., Schraml P. (2015). pVHL/HIF-regulated CD70 expression is associated with infiltration of CD27+ lymphocytes and increased serum levels of soluble CD27 in clear cell renal cell carcinoma. Clin. Cancer Res..

[28] Zhang H., Lu H., Xiang L., Bullen J.W., Zhang C., Samanta D., Gilkes D.M., He J., Semenza G.L. (2015). HIF-1 regulates CD47 expression in breast cancer cells to promote evasion of phagocytosis and maintenance of cancer stem cells. Proc. Natl. Acad. Sci. USA.

[29] Riether C., Schürch C.M., Flury C., Hinterbrandner M., Drück L., Huguenin A.L., Baerlocher G.M., Radpour R., Ochsenbein A.F. (2015). Tyrosine kinase inhibitor-induced CD70 expression mediates drug resistance in leukemia stem cells by activating Wnt signaling. Sci. Transl. Med..

[30] Li H., Huang C., Zhang Z., Feng Y., Wang Z., Tang X., Zhong K., Hu Y., Guo G., Zhou L. (2020). MEK inhibitor augments antitumor activity of B7-H3-redirected bispecific antibody. Front. Oncol..

[31] Glorieux C., Huang P. (2019). Regulation of CD137 expression through K-Ras signaling in pancreatic cancer cells. Cancer Commun..

[32] Kučan Brlić P., Lenac Roviš T., Cinamon G., Tsukerman P., Mandelboim O., Jonjić S. (2019). Targeting PVR (CD155) and its receptors in anti-tumor therapy. Cell. Mol. Immunol..

[33] Wang J., Wei W., Tang Q., Lu L., Luo Z., Li W., Lu Y., Pu J. (2020). Oxysophocarpine suppresses hepatocellular carcinoma growth and sensitizes the therapeutic blockade of anti-Lag-3 via reducing FGL1 expression. Cancer Med..

[34] Li R., Zatloukalova P., Muller P., Gil-Mir M., Kote S., Wilkinson S., Kemp A.J., Hernychova L., Wang Y., Ball K.L. (2020). The MDM2 ligand Nutlin-3 differentially alters expression of the immune blockade receptors PD-L1 and CD276. Cell. Mol. Biol. Lett..

[35] Keir M.E., Butte M.J., Freeman G.J., Sharpe A.H. (2008). PD-1 and its ligands in tolerance and immunity. Annu. Rev. Immunol..

[36] Sharma P., Allison J.P. (2015). The future of immune checkpoint therapy. Science.

[37] Ni L., Dong C. (2017). New B7 family checkpoints in human cancers. Mol. Cancer Ther..

[38] Marcucci F., Rumio C. (2021). Glycolysis-induced drug resistance in tumors-A response to danger signals?. Neoplasia.

[39] Zhi Y., Mou Z., Chen J., He Y., Dong H., Fu X., Wu Y. (2015). B7H1 expression and epithelial-to-mesenchymal transition phenotypes on colorectal cancer stem-like cells. PLoS ONE.

[40] Sanchez-Tillo E., Liu Y., De Barrios O., Siles L., Fanlo L., Cuatrecasas M., Darling D.S., Dean D.C., Castells A., Postigo A. (2012). EMT-activating transcription factors in cancer: Beyond EMT and tumor invasiveness. Cell. Mol. Life Sci..

[41] Dongre A., Weinberg R.A. (2019). New insights into the mechanisms of epithelial-mesenchymal transition and implications for cancer. Nat. Rev. Mol. Cell Biol..

[42] Marcucci F., Stassi G., De Maria R. (2016). Epithelial–mesenchymal transition: A new target in anticancer drug discovery. Nat. Rev. Drug Discov..

[43] Feng Y., Yang Z., Zhang C., Che N., Liu X., Xuan Y. (2021). B7-H4 induces epithelial-mesenchymal transition and promotes colorectal cancer stemness. Pathol. Res. Pract..

[44] Zheng Q., Gao J., Yin P., Wang W., Wang B., Li Y., Zhao C. (2020). CD155 contributes to the mesenchymal phenotype of triple-negative breast cancer. Cancer Sci..

[45] Ge Y., Chen W., Zhang X., Wang H., Cui J., Liu Y., Ju S., Tian X., Ju S. (2020). Nuclear-localized costimulatory molecule 4-1BBL promotes colon cancer cell proliferation and migration by regulating nuclear Gsk3β, and is linked to the poor outcomes associated with colon cancer. Cell Cycle.

[46] Zhang Y., Qiao H.X., Zhou Y.T., Hong L., Chen J.H. (2018). Fibrinogen-like-protein 1 promotes the invasion and metastasis of gastric cancer and is associated with poor prognosis. Mol. Med. Rep..

[47] Li J., Chen L., Xiong Y., Zheng X., Xie Q., Zhou Q., Shi L., Wu C., Jiang J., Wang H. (2017). Knockdown of PD-L1 in human gastric cancer cells inhibits tumor progression and improves the cytotoxic sensitivity to CIK therapy. Cell. Physiol. Biochem..

[48] Noman M.Z., Janji B., Abdou A., Hasmim M., Terry S., Tan T.Z., Mami-Chouaib F., Thiery J.P., Chouaib S. (2017). The immune checkpoint ligand PD-L1 is upregulated in EMT-activated human breast cancer cells by a mechanism involving ZEB-1 and miR-200. Oncoimmunology.

[49] Jiang B., Zhang T., Liu F., Sun Z., Shi H., Hua D., Yang C. (2016). The co-stimulatory molecule B7-H3 promotes the epithelial-mesenchymal transition in colorectal cancer. Oncotarget.

[50] Shan B., Man H., Liu J., Wang L., Zhu T., Ma M., Xv Z., Chen X., Yang X., Li P. (2016). TIM-3 promotes the metastasis of esophageal squamous cell carcinoma by targeting epithelial-mesenchymal transition via the Akt/GSK-3β/Snail signaling pathway. Oncol. Rep..

[51] Wang Y., Wang H., Zhao Q., Xia Y., Hu X., Guo J. (2015). PD-L1 induces epithelial-to-mesenchymal transition via activating SREBP-1c in renal cell carcinoma. Med. Oncol..

[52] Romeo E., Caserta C.A., Rumio C., Marcucci F. (2019). The vicious cross-talk between tumor cells with an EMT phenotype and cells of the immune system. Cells.

[53] Shields B.D., Koss B., Taylor E.M., Storey A.J., West K.L., Byrum S.D., Mackintosh S.G., Edmondson R., Mahmoud F., Shalin S.C. (2019). Loss of E-cadherin inhibits CD103 antitumor activity and reduces checkpoint blockade responsiveness in melanoma. Cancer Res..

[54] Mak M.P., Tong P., Diao L., Cardnell R.J., Gibbons D.L., William W.N., Skoulidis F., Parra E.R., Rodriguez-Canales J., Wistuba I.I. (2016). A patient-derived, pan-cancer EMT signature identifies global molecular alterations and immune target enrichment following epithelial-to-mesenchymal transition. Clin. Cancer Res..

[55] Lou Y., Diao L., Cuentas E.R.P., Denning W.L., Chen L., Fan Y.H., Byers L.A., Wang J., Papadimitrakopoulou V.A., Behrens C. (2016). Epithelial–mesenchymal transition is associated with a distinct tumor microenvironment including elevation of inflammatory signals and multiple immune checkpoints in lung adenocarcinoma. Clin. Cancer Res..

[56] Emran A.A., Chatterjee A., Rodger E.J., Tiffen J.C., Gallagher S.J., Eccles M.R., Hersey P. (2019). Reactive oxygen species modulate macrophage immunosuppressive phenotype through the up-regulation of PD-L1. Proc. Natl. Acad. Sci. USA.

[57] Ock C.Y., Kim S., Keam B., Kim M., Kim T.M., Kim J.H., Jeon Y.K., Lee J.S., Kwon S.K., Hah J.H. (2016). PD-L1 expression is associated with epithelial-mesenchymal transition in head and neck squamous cell carcinoma. Oncotarget.

[58] Xie N., Cai J.B., Zhang L., Zhang P.F., Shen Y.H., Yang X., Lu J.C., Gao D.M., Kang Q., Liu L.X. (2017). Upregulation of B7-H4 promotes tumor progression of intrahepatic cholangiocarcinoma. Cell Death Dis..

[59] Pai S., Bamodu O.A., Lin Y.K., Lin C.S., Chu P.Y., Chien M.H., Wang L.S., Hsiao M., Yeh C.T., Tsai J.T. (2019). CD47-SIRPα signaling induces epithelial-mesenchymal transition and cancer stemness and links to a poor prognosis in patients with oral squamous cell carcinoma. Cells.

[60] Noman M.Z., Van Moer K., Marani V., Gemmill R.M., Tranchevent L.C., Azuaje F., Muller A., Chouaib S., Thiery J.P., Berchem G. (2018). CD47 is a direct target of SNAI1 and ZEB1 and its blockade activates the phagocytosis of breast cancer cells undergoing EMT. Oncoimmunology.

[61] Zhao H., Wang J., Kong X., Li E., Liu Y., Du X., Kang Z., Tang Y., Kuang Y., Yang Z. (2016). CD47 promotes tumor invasion and metastasis in non-small cell lung cancer. Sci. Rep..

[62] Grimmig T., Gasser M., Moench R., Zhu L.J., Nawalaniec K., Callies S., Wagner M., Polat B., Mothi S.S., Luo Y. (2019). Expression of tumor-mediated CD137 ligand in human colon cancer indicates dual signaling effects. Oncoimmunology.

[63] Xiao Y., Qing J., Li B., Chen L., Nong S., Yang W., Tang X., Chen Z. (2020). TIM-3 participates in the invasion and metastasis of nasopharyngeal carcinoma via SMAD7/SMAD2/SNAIL1 axis-mediated epithelial-mesenchymal transition. OncoTargets Ther..

[64] Bertino P., Premeaux T.A., Fujita T., Haun B.K., Marciel M.P., Hoffmann F.W., Garcia A., Yiang H., Pastorino S., Carbone M. (2019). Targeting the C-terminus of galectin-9 induces mesothelioma apoptosis and M2 macrophage depletion. Oncoimmunology.

[65] Ge H., Mu L., Jin L., Yang C., Chang Y.E., Long Y., DeLeon G., Deleyrolle L., Mitchell D.A., Kubilis P.S. (2017). Tumor associated CD70 expression is involved in promoting tumor migration and macrophage infiltration in GBM. Int. J. Cancer.

[66] Pich C., Sarrabayrouse G., Teiti I., Mariamé B., Rochaix P., Lamant L., Favre G., Maisongrosse V., Tilkin-Mariamé A.F. (2016). Melanoma-expressed CD70 is involved in invasion and metastasis. Br. J. Cancer.

[67] Aggarwal S., He T., Fitzhugh W., Rosenthal K., Field B., Heidbrink J., Mesmer D., Ruben S.M., Moore P.A. (2009). Immune modulator CD70 as a potential cisplatin resistance predictive marker in ovarian cancer. Gynecol. Oncol..

[68] Budczies J., Bockmayr M., Denkert C., Klauschen F., Gröschel S., Darb-Esfahani S., Pfarr N., Leichsenring J., Onozato M.L., Lennerz J.K. (2016). Pan-cancer analysis of copy number changes in programmed death-ligand 1 (PD-L1, CD274)—Associations with gene expression, mutational load, and survival. Genes Chromosomes Cancer.

[69] Barrett M.T., Anderson K.S., Lenkiewicz E., Andreozzi M., Cunliffe H.E., Klassen C.L., Dueck A.C., McCullough A.E., Reddy S.K., Ramanathan R.K. (2015). Genomic amplification of 9p24.1 targeting JAK2, PD-L1, and PD-L2 is enriched in high-risk triple negative breast cancer. Oncotarget.

[70] Pastushenko I., Brisebarre A., Sifrim A., Fioramonti M., Revenco T., Boumahdi S., Van Keymeulen A., Brown D., Moers V., Lemaire S. (2018). Identification of the tumour transition states occurring during EMT. Nature.

[71] Calderaro J., Rousseau B., Amaddeo G., Mercey M., Charpy C., Costentin C., Luciani A., Zafrani E.S., Laurent A., Azoulay D. (2016). Programmed death ligand 1 expression in hepatocellular carcinoma: Relationship with clinical and pathological features. Hepatology.

[72] Madore J., Vilain R.E., Menzies A.M., Kakavand H., Wilmott J.S., Hyman J., Yearley J.H., Kefford R.F., Thompson J.F., Long G.V. (2015). PD-L1 expression in melanoma shows marked heterogeneity within and between patients: Implications for anti-PD-1/PD-L1 clinical trials. Pigment. Cell Melanoma Res..

[73] Lepletier A., Madore J., O’Donnell J.S., Johnston R.L., Li X.Y., McDonald E., Ahern E., Kuchel A., Eastgate M., Pearson S.A. (2020). Tumor CD155 expression is associated with resistance to anti-PD1 immunotherapy in metastatic melanoma. Clin. Cancer Res..

[74] Song X., Zhou Z., Li H., Xue Y., Lu X., Bahar I., Kepp O., Hung M.C., Kroemer G., Wan Y. (2020). Pharmacologic suppression of B7-H4 glycosylation restores antitumor immunity in immune-cold breast cancers. Cancer Discov..

[75] Cheng H., Borczuk A., Janakiram M., Ren X., Lin J., Assal A., Halmos B., Perez-Soler R., Zang X. (2018). Wide expression and significance of alternative immune checkpoint molecules, B7x and HHLA2, in PD-L1–negative human lung cancers. Clin. Cancer Res..

[76] Guo M., Yuan F., Qi F., Sun J., Rao Q., Zhao Z., Huang P., Fang T., Yang B., Xia J. (2020). Expression and clinical significance of LAG-3, FGL1, PD-L1 and CD8 ^+^ T cells in hepatocellular carcinoma using multiplex quantitative analysis. J. Transl. Med..

[77] Malissen N., Macagno N., Granjeaud S., Granier C., Moutardier V., Gaudy-Marqueste C., Habel N., Mandavit M., Guillot B., Pasero C. (2019). HVEM has a broader expression than PD-L1 and constitutes a negative prognostic marker and potential treatment target for melanoma. Oncoimmunology.

[78] Koyama S., Akbay E.A., Li Y.Y., Herter-Sprie G.S., Buczkowski K.A., Richards W.G., Gandhi L., Redig A.J., Rodig S.J., Asahina H. (2016). Adaptive resistance to therapeutic PD-1 blockade is associated with upregulation of alternative immune checkpoints. Nat. Commun..

[79] Stewart R., Morrow M., Hammond S.A., Mulgrew K., Marcus D., Poon E., Watkins A., Mullins S., Chodorge M., Andrews J. (2015). Identification and characterization of MEDI4736, an antagonistic anti-PD-L1 monoclonal antibody. Cancer Immunol. Res..

[80] Casella G., Rasouli J., Thome R., Descamps H.C., Vattikonda A., Ishikawa L., Boehm A., Hwang D., Zhang W., Xiao D. (2020). Interferon-γ/Interleukin-27 axis induces programmed death ligand 1 expression in monocyte-derived dendritic cells and restores immune tolerance in central nervous system autoimmunity. Front. Immunol..

[81] Zong Z., Zou J., Mao R., Ma C., Li N., Wang J., Wang X., Zhou H., Zhang L., Shi Y. (2019). M1 macrophages induce PD-L1 expression in hepatocellular carcinoma cells through IL-1β signaling. Front. Immunol..

[82] Shklovskaya E., Rizos H. (2020). spatial and temporal changes in PD-L1 expression in cancer: The role of genetic drivers, tumor microenvironment and resistance to therapy. Int. J. Mol. Sci..

[83] Lee Y., Moon Y., Hyung K., Yoo J. (2013). Macrophage PD-L1 strikes back: PD-1/PDL1 interaction drives macrophages toward regulatory subsets. Adv. Biosci..

[84] Herbst R.S., Soria J.C., Kowanetz M., Fine G.D., Hamid O., Gordon M.S., Sosman J.A., McDermott D.F., Powderly J.D., Gettinger S.N. (2014). Predictive correlates of response to the anti-PD-L1 antibody MPDL3280A in cancer patients. Nature.

[85] Kryczek I., Zou L., Rodriguez P., Zhu G., Wei S., Mottram P., Brumlik M., Cheng P., Curiel T., Myers L. (2006). B7-H4 expression identifies a novel suppressive macrophage population in human ovarian carcinoma. J. Exp. Med..

[86] Hao N.B., Lü M.H., Fan Y.H., Cao Y.L., Zhang Z.R., Yang S.M. (2012). Macrophages in tumor microenvironments and the progression of tumors. Clin. Dev. Immunol..

[87] Pulko V., Harris K.J., Liu X., Gibbons R.M., Harrington S.M., Krco C.J., Kwon E.D., Dong H. (2011). B7-h1 expressed by activated CD8 T cells is essential for their survival. J. Immunol..

[88] Liu X., Wu X., Cao S., Harrington S.M., Yin P., Mansfield A.S., Dong H. (2016). B7-H1 antibodies lose antitumor activity due to activation of p38 MAPK that leads to apoptosis of tumor-reactive CD8(+) T cells. Sci. Rep..

[89] Diskin B., Adam S., Cassini M.F., Sanchez G., Liria M., Aykut B., Buttar C., Li E., Sundberg B., Salas R.D. (2020). PD-L1 engagement on T cells promotes self-tolerance and suppression of neighboring macrophages and effector T cells in cancer. Nat. Immunol..

[90] Teramoto K., Igarashi T., Kataoka Y., Ishida M., Hanaoka J., Sumimoto H., Daigo Y. (2019). Clinical significance of PD-L1-positive cancer-associated fibroblasts in pN0M0 non-small cell lung cancer. Lung Cancer.

[91] Li X.Y., Das I., Lepletier A., Addala V., Bald T., Stannard K., Barkauskas D., Liu J., Aguilera A.R., Takeda K. (2018). CD155 loss enhances tumor suppression via combined host and tumor-intrinsic mechanisms. J. Clin. Investig..

[92] Juneja V.R., McGuire K.A., Manguso R.T., LaFleur M.W., Collins N., Haining W.N., Freeman G.J., Sharpe A.H. (2017). PD-L1 on tumor cells is sufficient for immune evasion in immunogenic tumors and inhibits CD8 T cell cytotoxicity. J. Exp. Med..

[93] Kleinovink J.W., van Hall T., Ossendorp F., Fransen M.F. (2017). PD-L1 immune suppression in cancer: Tumor cells or host cells?. Oncoimmunology.

[94] Dong W., Wu X., Ma S., Wang Y., Nalin A.P., Zhu Z., Zhang J., Benson D.M., He K., Caligiuri M.A. (2019). The mechanism of anti-PD-L1 antibody efficacy against PD-L1-negative tumors identifies NK cells expressing PD-L1 as a cytolytic effector. Cancer Discov..

[95] Champiat S., Dercle L., Ammari S., Massard C., Hollebecque A., Postel-Vinay S., Chaput N., Eggermont A., Marabelle A., Soria J.C. (2017). Hyperprogressive disease is a new pattern of progression in cancer patients treated by anti-PD-1/PDL1. Clin. Cancer Res..

[96] Ferrara R., Mezquita L., Texier M., Lahmar J., Audigier-Valette C., Tessonnier L., Mazieres J., Zalcman G., Brosseau S., Le Moulec S. (2018). Hyperprogressive disease in patients with advanced non-small cell lung cancer treated with PD-1/PD-L1 inhibitors or with single-agent chemotherapy. JAMA Oncol..

[97] Marcucci F., Rumio C. (2021). The tumor-promoting effects of the adaptive immune system: A cause of hyperprogressive disease in cancer?. Cell. Mol. Life Sci..

[98] Daud A.I., Wolchok J.D., Robert C., Hwu W.J., Weber J.S., Ribas A., Hodi F.S., Joshua A.M., Kefford R., Hersey P. (2016). Programmed death-ligand 1 expression and response to the anti-programmed death 1 antibody pembrolizumab in melanoma. J. Clin. Oncol..

[99] Robert C., Long G.V., Brady B., Dutriaux C., Maio M., Mortier L., Hassel J.C., Rutkowski P., McNeil C., Kalinka-Warzocha E. (2014). Nivolumab in previously untreated melanoma without BRAF mutation. N. Engl. J. Med..

[100] Taube J.M., Klein A., Brahmer J.R., Xu H., Pan X., Kim J.H., Chen L., Pardoll D.M., Topalian S.L., Anders R.A. (2014). Association of PD-1, PD-1 ligands, and other features of the tumor immune microenvironment with response to anti-PD-1 therapy. Clin. Cancer Res..

[101] Dahan R., Sega E., Engelhardt J., Selby M., Korman A.J., Ravetch J.V. (2015). FcγRs modulate the anti-tumor activity of antibodies targeting the PD-1/PD-L1 axis. Cancer Cell.

[102] Boyerinas B., Jochems C., Fantini M., Heery C.R., Gulley J.L., Tsang K., Schlom J. (2015). Antibody-dependent cellular cytotoxicity activity of a novel anti-PD-L1 antibody avelumab (MSB0010718C) on human tumor cells. Cancer Immunol. Res..

[103] Khanna S., Thomas A., Abate-Daga D., Zhang J., Morrow B., Steinberg S.M., Orlandi A., Ferroni P., Schlom J., Guadagni F. (2016). Malignant mesothelioma effusions are infiltrated by CD3 ^+^ T Cells highly expressing PD-L1 and the PD-L1 ^+^ tumor cells within these effusions are susceptible to ADCC by the anti-PD-L1 antibody avelumab. J. Thorac. Oncol..

[104] Giles A.J., Hao S., Padget M., Song H., Zhang W., Lynes J., Sanchez V., Liu Y., Jung J., Cao X. (2019). Efficient ADCC killing of meningioma by avelumab and a high-affinity natural killer cell line, haNK. JCI Insight.

[105] Hamilton G., Rath B. (2017). Avelumab: Combining immune checkpoint inhibition and antibody-dependent cytotoxicity. Expert Opin. Biol. Ther..

[106] Byrd J.C., Kipps T.J., Flinn I.W., Cooper M., Odenike O., Bendiske J., Rediske J., Bilic S., Dey J., Baeck J. (2012). Phase I study of the anti-CD40 humanized monoclonal antibody lucatumumab (HCD122) in relapsed chronic lymphocytic leukemia. Leuk. Lymphoma.

[107] Kim M.J., Lee J.C., Lee J.J., Kim S., Lee S.G., Park S.W., Sung M.W., Heo D.S. (2008). Association of CD47 with natural killer cell-mediated cytotoxicity of head-and-neck squamous cell carcinoma lines. Tumour Biol..

[108] Gao Y., Zhang D., Yang C., Duan X., Li X., Zhong D. (2019). Two validated liquid chromatography-mass spectrometry methods with different pretreatments for the quantification of an anti-CD47 monoclonal antibody in rat and cynomolgus monkey serum compared with an electrochemiluminescence method. J. Pharm. Biomed. Anal..

[109] Park J.E., Kim S.E., Keam B., Park H.R., Kim S., Kim M., Kim T.M., Doh J., Kim D.W., Heo D.S. (2020). Anti-tumor effects of NK cells and anti-PD-L1 antibody with antibody-dependent cellular cytotoxicity in PD-L1-positive cancer cell lines. J. Immunother. Cancer.

[110] McEarchern J.A., Smith L.M., McDonagh C.F., Klussman K., Gordon K.A., Morris-Tilden C.A., Duniho S., Ryan M., Boursalian T.E., Carter P.J. (2008). Preclinical characterization of SGN-70, a humanized antibody directed against CD70. Clin. Cancer Res..

[111] Jeon H., Vigdorovich V., Garrett-Thomson S.C., Janakiram M., Ramagopal U.A., Abadi Y.M., Lee J.S., Scandiuzzi L., Ohaegbulam K.C., Chinai J.M. (2014). Structure and cancer immunotherapy of the B7 family member B7x. Cell Rep..

[112] Dong H., Strome S.E., Salomao D.R., Tamura H., Hirano F., Flies D.B., Roche P.C., Lu J., Zhu G., Tamada K. (2002). Tumor-associated B7-H1 promotes T-cell apoptosis: A potential mechanism of immune evasion. Nat. Med..

[113] Ju X., Zhang H., Zhou Z., Chen M., Wang Q. (2020). Tumor-associated macrophages induce PD-L1 expression in gastric cancer cells through IL-6 and TNF-α signaling. Exp. Cell Res..

[114] Zhou X., Mao Y., Zhu J., Meng F., Chen Q., Tao L., Li R., Fu F., Liu C., Hu Y. (2016). TGF-β1 promotes colorectal cancer immune escape by elevating B7-H3 and B7-H4 via the miR-155/miR-143 axis. Oncotarget.

[115] Kryczek I., Wei S., Zou L., Zhu G., Mottram P., Xu H., Chen L., Zou W. (2006). induction of B7-H4 on APCs through IL-10: Novel suppressive mode for regulatory T cells. J. Immunol..

[116] Corti A., Curnis F., Rossoni G., Marcucci F., Gregorc V. (2013). Peptide-mediated targeting of cytokines to tumor vasculature: The NGR-hTNF example. BioDrugs.

[117] Pasche N., Wulhfard S., Pretto F., Carugati E., Neri D. (2012). The antibody-based delivery of interleukin-12 to the tumor neovasculature eradicates murine models of cancer in combination with paclitaxel. Clin. Cancer Res..

[118] Vilain R.E., Menzies A.M., Wilmott J.S., Kakavand H., Madore J., Guminski A., Liniker E., Kong B.Y., Cooper A.J., Howle J.R. (2017). Dynamic changes in PD-L1 expression and immune infiltrates early during treatment predict response to PD-1 blockade in melanoma. Clin. Cancer Res..

[119] Yamazaki T., Akiba H., Koyanagi A., Azuma M., Yagita H., Okumura K. (2005). Blockade of B7-H1 on macrophages suppresses CD4^+^ T cell proliferation by augmenting IFN-gamma-induced nitric oxide production. J. Immunol..

[120] Marcucci F., Bellone M., Caserta C.A., Corti A. (2014). Pushing tumor cells towards a malignant phenotype. Stimuli from the microenvironment, intercellular communications and alternative roads. Int. J. Cancer.

[121] Yang M., Liu P., Wang K., Glorieux C., Hu Y., Wen S., Jiang W., Huang P. (2017). Chemotherapy induces tumor immune evasion by upregulation of programmed cell death ligand 1 expression in bone marrow stromal cells. Mol. Oncol..

[122] Niu C., Jin H., Li M., Zhu S., Zhou L., Jin F., Zhou Y., Xu D., Xu J., Zhao L. (2017). Low-dose bortezomib increases the expression of NKG2D and DNAM-1 ligands and enhances induced NK and γδ T cell-mediated lysis in multiple myeloma. Oncotarget.

[123] López-Cobo S., Pieper N., Campos-Silva C., García-Cuesta E.M., Reyburn H.T., Paschen A., Valés-Gómez M. (2018). Impaired NK cell recognition of vemurafenib-treated melanoma cells is overcome by simultaneous application of histone deacetylase inhibitors. Oncoimmunology.

[124] Sheng J., Fang W., Yu J., Chen N., Zhan J., Ma Y., Yang Y., Yan H., Zhao H., Zhang L. (2016). Expression of programmed death ligand-1 on tumor cells varies pre and post chemotherapy in non-small cell lung cancer. Sci. Rep..

[125] Zhang J., Bu X., Wang H., Zhu Y., Geng Y., Nihira N.T., Tan Y., Ci Y., Wu F., Dai X. (2018). Cyclin D-CDK4 kinase destabilizes PD-L1 via cullin 3-SPOP to control cancer immune surveillance. Nature.

[126] Goel S., DeCristo M.J., Watt A.C., BrinJones H., Sceneay J., Li B.B., Khan N., Ubellacker J.M., Xie S., Metzger-Filho O. (2017). CDK4/6 inhibition triggers anti-tumour immunity. Nature.

[127] Emran A.A., Chatterjee A., Rodger E.J., Tiffen J.C., Gallagher S.J., Eccles M.R., Hersey P. (2019). Targeting DNA methylation and EZH2 Activity to Overcome Melanoma Resistance to Immunotherapy. Trends Immunol..

[128] Iwasa M., Harada T., Oda A., Bat-Erdene A., Teramachi J., Tenshin H., Ashtar M., Oura M., Sogabe K., Udaka K. (2019). PD-L1 upregulation in myeloma cells by panobinostat in combination with interferon-γ. Oncotarget.

[129] Zhou S., Zhao X., Yang Z., Yang R., Chen C., Zhao K., Wang W., Ma Y., Zhang Q., Wang X. (2019). Neddylation inhibition upregulates PD-L1 expression and enhances the efficacy of immune checkpoint blockade in glioblastoma. Int. J. Cancer.

[130] Loo D., Alderson R.F., Chen F.Z., Huang L., Zhang W., Gorlatov S., Burke S., Ciccarone V., Li H., Yang Y. (2012). Development of an Fc-enhanced anti-B7-H3 monoclonal antibody with potent antitumor activity. Clin. Cancer Res..

[131] Seaman S., Zhu Z., Saha S., Zhang X.M., Yang M.Y., Hilton M.B., Morris K., Szot C., Morris H., Swing D.A. (2017). Eradication of tumors through simultaneous ablation of CD276/B7-H3-positive tumor cells and tumor vasculature. Cancer Cell.

[132] Du H., Hirabayashi K., Ahn S., Kren N.P., Montgomery S.A., Wang X., Tiruthani K., Mirlekar B., Michaud D., Greene K. (2019). Antitumor responses in the absence of toxicity in solid tumors by targeting B7-H3 via chimeric antigen receptor T cells. Cancer Cell.

[133] Zhan S., Liu Z., Zhang M., Guo T., Quan Q., Huang L., Guo L., Cao L., Zhang X. (2020). Overexpression of B7-H3 in α-SMA-positive fibroblasts is associated with cancer progression and survival in gastric adenocarcinomas. Front. Oncol..

[134] Dutsch-Wicherek M., Kazmierczak W. (2013). Creation of a suppressive microenvironment by macrophages and cancer-associated fibroblasts. Front. Biosci..

[135] Aftimos P., Rolfo C., Rottey S., Offner F., Bron D., Maerevoet M., Soria J.C., Moshir M., Dreier T., Van Rompaey L. (2017). Phase I Dose-escalation study of the anti-CD70 antibody ARGX-110 in advanced malignancies. Clin. Cancer Res..

[136] Hermans C., Rolfo C., Peeters M., De Wever O., Lardon F., Siozopoulou V., Smits E., Pauwels P. (2018). Unveiling a CD70-positive subset of cancer-associated fibroblasts marked by pro-migratory activity and thriving regulatory T cell accumulation. Oncoimmunology.

[137] De Meulenaere A., Vermassen T., Aspeslagh S., Zwaenepoel K., Deron P., Duprez F., Ferdinande L., Rottey S. (2016). CD70 expression and its correlation with clinicopathological variables in squamous cell carcinoma of the head and neck. Pathobiology.

[138] Escalante N.K., von Rossum A., Lee M., Choy J.C. (2011). CD155 on human vascular endothelial cells attenuates the acquisition of effector functions in CD8 T cells. Arterioscler. Thromb. Vasc Biol..

[139] Thomas L.J., Vitale L., O’Neill T., Dolnick R.Y., Wallace P.K., Minderman H., Gergel L.E., Forsberg E.M., Boyer J.M., Storey J.R. (2016). Development of a novel antibody-drug conjugate for the potential treatment of ovarian, lung, and renal cell carcinoma expressing TIM-1. Mol. Cancer Ther..

[140] Kaur S., Cicalese K.V., Bannerjee R., Roberts D.D. (2020). Preclinical and clinical development of therapeutic antibodies targeting functions of CD47 in the tumor microenvironment. Antib. Ther..

[141] Griffioen A.W., Thijssen V.L. (2014). Galectins in tumor angiogenesis. Ann. Transl. Med..

[142] Lewis T.S., McCormick R.S., Stone I.J., Emmerton K., Mbow B., Miyamoto J., Drachman J.G., Grewal I.S., Law C.L. (2011). Proapoptotic signaling activity of the anti-CD40 monoclonal antibody dacetuzumab circumvents multiple oncogenic transformation events and chemosensitizes NHL cells. Leukemia.

[143] Kluth B., Hess S., Engelmann H., Schafnitzel S., Riethmüller G., Feucht H.E. (1997). Endothelial expression of CD40 in renal cell carcinoma. Cancer Res..

[144] Silence K., Dreier T., Moshir M., Ulrichts P., Gabriels S.M., Saunders M., Wajant H., Brouckaert P., Huyghe L., Van Hauwermeiren T. (2014). ARGX-110, a highly potent antibody targeting CD70, eliminates tumors via both enhanced ADCC and immune checkpoint blockade. mAbs.

[145] Oflazoglu E., Stone I.J., Gordon K., Wood C.G., Repasky E.A., Grewal I.S., Law C.-L., Gerber H.-P. (2008). Potent anticarcinoma activity of the humanized anti-CD70 antibody h1F6 conjugated to the tubulin inhibitor auristatin via an uncleavable linker. Clin. Cancer Res..

[146] Tannir N.M., Forero-Torres A., Ramchandren R., Pal S.K., Ansell S.M., Infante J.R., de Vos S., Hamlin P.A., Kim S.K., Whiting N.C. (2014). Phase I dose-escalation study of SGN-75 in patients with CD70-positive relapsed/refractory non-Hodgkin lymphoma or metastatic renal cell carcinoma. Investig. New Drugs.

[147] Jeffrey S.C., Burke P.J., Lyon R.P., Meyer D.W., Sussman D., Anderson M., Hunter J.H., Leiske C.I., Miyamoto J.B., Nicholas N.D. (2013). A potent anti-CD70 antibody−drug conjugate combining a dimeric pyrrolobenzodiazepine drug with site-specific conjugation technology. Bioconjugate Chem..

[148] Phillips T., Barr P.M., Park S.I., Kolibaba K., Caimi P.F., Chhabra S., Kingsley E.C., Boyd T., Chen R., Carret A.S. (2019). A phase 1 trial of SGN-CD70A in patients with CD70-positive diffuse large B cell lymphoma and mantle cell lymphoma. Investig. New Drugs.

[149] Wang H., Rangan V.S., Sung M.C., Passmore D., Kempe T., Wang X., Thevanayagam L., Pan C., Rao C., Srinivasan M. (2016). Pharmacokinetic characterization of BMS-936561, an anti-CD70 antibody-drug conjugate, in preclinical animal species and prediction of its pharmacokinetics in humans. Biopharm. Drug Dispos..

[150] Owonikoko T.K., Hussain A., Stadler W.M., Smith D.C., Kluger H., Molina A.M., Gulati P., Shah A., Ahlers C.M., Cardarelli P.M. (2016). First-in-human multicenter phase I study of BMS-936561 (MDX-1203), an antibody-drug conjugate targeting CD70. Cancer Chemother. Pharmacol..

[151] Scribner J.A., Brown J.G., Son T., Chiechi M., Li P., Sharma S., Li H., De Costa A., Li Y., Chen Y. (2020). Preclinical development of MGC018, a duocarmycin-based antibody-drug conjugate targeting B7-H3 for solid cancer. Mol. Cancer Ther..

[152] Zhu L., Liu J., Zhou G., Ng H.M., Ang I.L., Ma G., Liu Y., Yang S., Zhang F., Miao K. (2019). Targeting immune checkpoint B7-H3 antibody-chlorin e6 bioconjugates for spectroscopic photoacoustic imaging and photodynamic therapy. Chem. Commun..

[153] Leong S.R., Liang W.C., Wu Y., Crocker L., Cheng E., Sampath D., Ohri R., Raab H., Hass P.E., Pham T. (2015). An anti-B7-H4 antibody-drug conjugate for the treatment of breast cancer. Mol. Pharm..

[154] Li C.W., Lim S.O., Chung E.M., Kim Y.S., Park A.H., Yao J., Cha J.H., Xia W., Chan L.C., Kim T. (2018). Eradication of triple-negative breast cancer cells by targeting glycosylated PD-L1. Cancer Cell.

[155] Kalim M., Wang S., Liang K., Khan M.S.I., Zhan J. (2020). Engineered scPDL1-DM1 drug conjugate with improved in vitro analysis to target PD-L1 positive cancer cells and intracellular trafficking studies in cancer therapy. Genet. Mol. Biol..

[156] Sau S., Petrovici A., Alsaab H.O., Bhise K., Iyer A.K. (2019). PDL-1 antibody drug conjugate for selective chemo-guided immune modulation of cancer. Cancers.

[157] Sun X., Yu Y., Ma L., Xue X., Gao Z., Ma J., Zhang M. (2020). T cell cytotoxicity toward hematologic malignancy via B7-H3 targeting. Investig. New Drugs.

[158] Zheng M., Yu L., Hu J., Zhang Z., Wang H., Lu D., Tang X., Huang J., Zhong K., Wang Z. (2020). Efficacy of B7-H3-redirected BiTE and CAR-T immunotherapies against extranodal nasal natural killer/T cell lymphoma. Transl. Oncol..

[159] Iizuka A., Nonomura C., Ashizawa T., Kondou R., Ohshima K., Sugino T., Mitsuya K., Hayashi N., Nakasu Y., Maruyama K. (2019). A T-cell-engaging B7-H4/CD3-bispecific Fab-scFv antibody targets human breast cancer. Clin. Cancer Res..

[160] Zhao H., Ma J., Lei T., Ma W., Zhang M. (2019). The bispecific anti-CD3 × anti-CD155 antibody mediates T cell immunotherapy for human prostate cancer. Investig. New Drugs.

[161] Ma W., Ma J., Lei T., Zhao M., Zhang M. (2019). Targeting immunotherapy for bladder cancer by using anti-CD3 × CD155 bispecific antibody. J. Cancer..

[162] Yang C.Y., Fan M.H., Miao C.H., Liao Y.J., Yuan R.H., Liu C.L. (2020). Engineering chimeric antigen receptor T cells against immune checkpoint inhibitors PD-1/PD-L1 for treating pancreatic cancer. Mol. Ther. Oncolytics.

[163] Huang B., Luo L., Wang J., He B., Feng R., Xian N., Zhang Q., Chen L., Huang G. (2019). B7-H3 specific T cells with chimeric antigen receptor and decoy PD-1 receptors eradicate established solid human tumors in mouse models. Oncoimmunology.

[164] Majzner R.G., Theruvath J.L., Nellan A., Heitzeneder S., Cui Y., Mount C.W., Rietberg S.P., Linde M.H., Xu P., Rota C. (2019). CAR T cells targeting B7-H3, a pan-cancer antigen, demonstrate potent preclinical activity against pediatric solid tumors and brain tumors. Clin. Cancer Res..

[165] Yang S., Cao B., Zhou G., Zhu L., Wang L., Zhang L., Kwok H.F., Zhang Z., Zhao Q. (2020). Targeting B7-H3 immune checkpoint with chimeric antigen receptor-engineered natural killer cells exhibits potent cytotoxicity against non-small cell lung cancer. Front. Pharmacol..

[166] Smith J.B., Lanitis E., Dangaj D., Buza E., Poussin M., Stashwick C., Scholler N., Powell D.J. (2016). Tumor regression and delayed onset toxicity following B7-H4 CAR T cell therapy. Mol. Ther..

[167] Golubovskaya V., Berahovich R., Zhou H., Xu S., Harto H., Li L., Chao C.C., Mao M.M., Wu L. (2017). CD47-CAR-T cells effectively kill target cancer cells and block pancreatic tumor growth. Cancers.

[168] Jin L., Ge H., Long Y., Yang C., Chang Y.E., Mu L., Sayour E.J., De Leon G., Wang Q.J., Yang J.C. (2018). CD70, a novel target of CAR T-cell therapy for gliomas. Neuro Oncol..

[169] Park Y.P., Jin L., Bennett K.B., Wang D., Fredenburg K.M., Tseng J.E., Chang L.J., Huang J., Chan E.K.L. (2018). CD70 as a target for chimeric antigen receptor T cells in head and neck squamous cell carcinoma. Oral Oncol..

[170] Shaffer D.R., Savoldo B., Yi Z., Chow K.K., Kakarla S., Spencer D.M., Dotti G., Wu M.F., Liu H., Kenney S. (2011). T cells redirected against CD70 for the immunotherapy of CD70-positive malignancies. Blood.

[171] Puro R.J., Bouchlaka M.N., Hiebsch R.R., Capoccia B.J., Donio M.J., Manning P.T., Frazier W.A., Karr R.W., Pereira D.S. (2020). Development of AO-176, a next-generation humanized anti-CD47 antibody with novel anticancer properties and negligible red blood cell binding. Mol. Cancer Ther..

[172] Pettersen R.D., Hestdal K., Olafsen M.K., Lie S.O., Lindberg F.P. (1999). CD47 signals T cell death. J. Immunol..

[173] Leclair P., Liu C.C., Monajemi M., Reid G.S., Sly L.M., Lim C.J. (2018). CD47-ligation induced cell death in T-acute lymphoblastic leukemia. Cell Death Dis..

[174] Cioffi M., Trabulo S., Hidalgo M., Costello E., Greenhalf W., Erkan M., Kleeff J., Sainz B., Heeschen C. (2015). Inhibition of CD47 effectively targets pancreatic cancer stem cells via dual mechanisms. Clin. Cancer Res..

[175] Wu X., Li F., Li Y., Yu Y., Liang C., Zhang B., Zhao C., Lu A., Zhang G. (2020). A PD-L1 aptamer selected by loss-gain cell-SELEX conjugated with paclitaxel for treating triple-negative breast cancer. Med. Sci. Monit..

[176] Gromeier M., Alexander L., Wimmer E. (1996). Internal ribosomal entry site substitution eliminates neurovirulence in intergeneric poliovirus recombinants. Proc. Natl Acad. Sci. USA.

[177] Brown M.C., Gromeier M. (2015). Cytotoxic and immunogenic mechanisms of recombinant oncolytic poliovirus. Curr. Opin. Virol..

[178] Desjardins A., Gromeier M., Herndon J.E., Beaubier N., Bolognesi D.P., Friedman A.H., Friedman H.S., McSherry F., Muscat A.M., Nair S. (2018). Recurrent glioblastoma treated with recombinant poliovirus. N. Engl. J. Med..

[179] Gerdes C.A., Nicolini V.G., Herter S., van Puijenbroek E., Lang S., Roemmele M., Moessner E., Freytag O., Friess T., Ries C.H. (2013). GA201 (RG7160): A novel, humanized, glycoengineered anti-EGFR antibody with enhanced ADCC and superior in vivo efficacy compared with cetuximab. Clin. Cancer Res..

[180] Ni X., Jorgensen J.L., Goswami M., Challagundla P., Decker W.K., Kim Y.H., Duvic M.A. (2015). Reduction of regulatory T cells by Mogamulizumab, a defucosylated anti-CC chemokine receptor 4 antibody, in patients with aggressive/refractory mycosis fungoides and Sézary syndrome. Clin. Cancer Res..

[181] Stavenhagen J.B., Gorlatov S., Tuaillon N., Rankin C.T., Li H., Burke S., Huang L., Vijh S., Johnson S., Bonvini E. (2007). Fc optimization of therapeutic antibodies enhances their ability to kill tumor cells in vitro and controls tumor expansion in vivo via low-affinity activating Fcgamma receptors. Cancer Res..

[182] Dan N., Setua S., Kashyap V.K., Khan S., Jaggi M., Yallapu M.M., Chauhan S.C. (2018). Antibody-drug conjugates for cancer therapy: Chemistry to clinical implications. Pharmaceuticals.

[183] Diamantis N., Banerji U. (2016). Antibody-drug conjugates—An emerging class of cancer treatment. Br. J. Cancer.

[184] Labrijn A.F., Janmaat M.L., Reichert J.M., Parren P.W.H.I. (2019). Bispecific antibodies: A mechanistic review of the pipeline. Nat. Rev. Drug Discov..

[185] Larson R.C., Maus M.V. (2021). Recent advances and discoveries in the mechanism and function of CAR T cells. Nat. Rev. Cancer.

[186] Wang H., Kaur G., Sankin A.I., Chen F., Guan F., Zang X. (2019). Immune checkpoint blockade and CAR-T cell therapy in hematologic malignancies. J. Hematol. Oncol..

[187] Zhou J., Rossi J. (2017). Aptamers as targeted therapeutics: Current potential and challenges. Nat. Rev. Drug Discov..

[188] Marcucci F., Corti A. (2012). Improving drug penetration to curb tumor drug resistance. Drug Discov. Today.

[189] Marcucci F., Corti A. (2012). How to improve exposure of tumor cells to drugs—Promoter drugs increase tumor uptake and penetration of effector drugs. Adv. Drug Deliv. Rev..

[190] Marcucci F., Bellone M., Rumio C., Corti A. (2013). Approaches to improve tumor accumulation and interactions between monoclonal antibodies and immune cells. mAbs.

[191] Perdigoto A.L., Kluger H., Herold K.C. (2021). Adverse events induced by immune checkpoint inhibitors. Curr. Opin. Immunol..

[192] Macek Jilkova Z., Aspord C., Kurma K., Granon A., Sengel C., Sturm N., Marche P.N., Decaens T. (2019). Immunologic features of patients with advanced hepatocellular carcinoma before and during sorafenib or anti-programmed death-1/programmed death-L1 treatment. Clin. Transl. Gastroenterol..

[193] Finn R.S., Qin S., Ikeda M., Galle P.R., Ducreux M., Kim T.Y., Kudo M., Breder V., Merle P., Kaseb A.O. (2020). Atezolizumab plus bevacizumab in unresectable hepatocellular carcinoma. N. Engl. J. Med..

[194] Rini B.I., Powles T., Atkins M.B., Escudier B., McDermott D.F., Suarez C., Bracarda S., Stadler W.M., Donskov F., Lee J.L. (2019). Atezolizumab plus bevacizumab versus sunitinib in patients with previously untreated metastatic renal cell carcinoma (IMmotion151): A multicentre, open-label, phase 3, randomised controlled trial. Lancet.

[195] Lan Y., Zhang D., Xu C., Hance K.W., Marelli B., Qi J., Yu H., Qin G., Sircar A., Hernández V.M. (2018). Enhanced preclinical antitumor activity of M7824, a bifunctional fusion protein simultaneously targeting PD-L1 and TGF-β. Sci. Transl. Med..

[196] Mariathasan S., Turley S.J., Nickles D., Castiglioni A., Yuen K., Wang Y., Kadel E.E., Koeppen H., Astarita J.L., Cubas R. (2018). TGFβ attenuates tumour response to PD-L1 blockade by contributing to exclusion of T cells. Nature.

